# Direct Extracellular Matrix Modulation Attenuates Intestinal Fibrosis via a Fibronectin‐Targeted Approach

**DOI:** 10.1002/advs.202519433

**Published:** 2026-01-30

**Authors:** Wenlong Ma, Siyu Yang, Tengkai Wang, Di Zhang, Hewei Wu, Shichen Fu, Xiaohan Wan, Lixiang Li, Xiuli Zuo, Yanqing Li, Jiaoyang Lu

**Affiliations:** ^1^ Department of Gastroenterology Qilu Hospital of Shandong University Jinan Shandong P. R. China; ^2^ Department of Medical Oncology Qilu Hospital of Shandong University Jinan Shandong P. R. China; ^3^ Laboratory of Translational Gastroenterology Qilu Hospital of Shandong University Jinan Shandong P. R. China; ^4^ Shandong Provincial Clinical Research Center for Digestive Disease Jinan Shandong P. R. China; ^5^ Medical Integration and Practice Center Shandong University Jinan Shandong P. R. China

**Keywords:** Crohn's disease, extracellular matrix, fibronectin, fibrosis

## Abstract

Intestinal fibrosis can progress independently of inflammation, driven by a self‐perpetuating cycle of extracellular matrix (ECM)‐myofibroblast interactions. However, due to the lack of reliable therapeutic targets within the ECM, the current strategy predominantly focuses on intracellular aspects of myofibroblasts, neglecting the regulation of the ECM itself. In this study, we first performed matrisomic analysis on human (ileal/colonic) and animal (decellularized/native) intestines, identifying fibronectin as the only ECM component consistently elevated in fibrotic versus normal gut tissues across all conditions. Subsequently, immunofluorescence co‐staining identified fibronectin as the principal structural scaffold of the fibrotic intestinal ECM. Furthermore, fibroblast‐specific Fn1 ablation ameliorates intestinal fibrosis and transforms refractory fibrotic thickening into reversible inflammatory thickening in both innate and adaptive immune‐driven models. Mechanistically, domain‐specific inhibitors (pUR4, polymerization inhibition; R1R2, collagen binding inhibition; ATN161, integrin engagement inhibition), combined with ECM‐mimetic platforms, demonstrated that fibronectin blockade directly inhibited its matrix assembly and impaired subsequent collagen fibrillogenesis—the major deposited component in fibrosis. Additionally, fibronectin‐depleted ECM diminished α5β1 integrin‐mediated mechanotransduction, thereby suppressing fibroblast activation and disrupting the self‐perpetuating cycle of intestinal fibrosis. Thus, fibronectin inhibition directly impedes ECM accumulation and ameliorates intestinal fibrosis, offering a new dimension for therapeutic intervention and an immediately druggable target in fibrotic diseases.

## Graphical abstract

Fibronectin regulates the extracellular matrix (ECM)–myofibroblast cycle through three key steps: (1) its secretion and assembly by myofibroblasts (inhibited by pUR4); (2) collagen deposition along its scaffold (inhibited by R1R2); and (3) integrin α5β1‐mediated mechanotransduction (inhibited by ATN161). Inhibiting this cycle disrupts fibrosis and improves intestinal fibrosis.

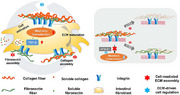

## 1 Introduction

Intestinal fibrosis represents a major complication of inflammatory bowel disease (IBD), particularly Crohn's disease. Currently, no effective pharmacological interventions exist; instead, only surgery provides temporary relief [1, 2]. Therefore, identifying effective anti‐intestinal fibrosis agents is critically important [3].

Conventional anti‐inflammatory therapies demonstrate limited efficacy in halting intestinal fibrosis progression. Importantly, a cumulating evidence indicates that fibrosis may persist and advance independently of active inflammation, driven by a self‐perpetuating extracellular matrix (ECM)‐myofibroblast (highly activated fibroblast) loop [2]. Within this pathological cycle, activated myofibroblasts excessively deposit pathological ECM, while the resultant stiff fibrotic ECM further promotes fibroblast activation through mechano‐biochemical signaling—establishing an inflammation‐independent pathogenic driver [1, 4, 5]. Notably, current anti‐fibrotic strategies targeting non‐inflammatory pathways predominantly focus on intracellular signaling mediators [6, 7], yet fail to adequately recognize the ECM's pivotal role as a therapeutic target capable of disrupting this vicious cycle. Crucially, neglecting interventions against dynamic ECM formation perpetuates the self‐amplifying fibrotic cascade. We therefore propose direct targeting of ECM assembly—with particular emphasis on fibronectin given its promising therapeutic potential—to inhibit ECM accumulation, interrupt this self‐reinforcing cycle, and ameliorate intestinal fibrosis.

Fibronectin constitutes an essential structural component of the ECM, forming integral networks with collagen (the predominant ECM constituent), fibrin, and other matrix proteins [8, 9]. Historically, Fibronectin was perceived as a terminal, stable, and biologically inert protein deposited during fibrosis—an understanding that established it as a reliable indicator of fibrosis severity [7, 10, 11]. However, emerging evidence suggests that fibronectin functions not merely as an inert structural component of the ECM, but rather as an active regulator of ECM dynamics [12]. Mechanistically, fibronectin networks assemble prior to collagen deposition, and their collagen‐binding domains significantly lower the concentration of free collagen monomers required for fibrillogenesis, thereby accelerating ECM accumulation. Furthermore, fibronectin scaffolds are crucial for the maturation of procollagen into collagen fibrils. This process is essential because the subsequent assembly of mature collagen into organized networks is severely impaired in fibronectin‐deficient microenvironments [13, 14, 15]. These findings suggest the potential to alleviate fibrosis by inhibiting fibronectin to halt the accumulation of ECM.

Fibronectin inhibition has shown anti‐fibrotic efficacy in mouse models of hepatic and cardiac fibrosis [16, 17]. However, limitations, including a limited understanding of ECM formation mechanisms and the absence of ECM‐mimetic models, have restricted these studies. Consequently, they were able only to demonstrate fibronectin inhibition's anti‐fibrotic efficacy, failing to elucidate its mechanistic basis. Additionally, intestinal fibrosis differs from cardiac fibrosis in etiology (inflammation vs ischemia) and from liver fibrosis in anatomy (hollow vs parenchymal organ). Consequently, the role and mechanism of fibronectin in intestinal fibrosis remain to be elucidated.

In this study, we identified fibronectin as the critical structural ‘scaffold’ of the ECM through proteomics of decellularized intestines and immunofluorescence. Subsequently, genetic ablation of Fn1 in two mouse models demonstrated that fibronectin inhibition ameliorated fibrosis and reduced aberrant ECM accumulation. By targeting key functional domains of fibronectin, we elucidated its regulatory mechanism in ECM organization and identified clinically translatable inhibitors. Furthermore, the development of novel fibronectin ECM‐mimetic models enabled us to investigate how fibronectin in the ECM influences intestinal fibroblast activation and the underlying mechanisms. In summary, we propose direct ECM modulation as a novel therapeutic strategy for alleviating intestinal fibrosis and identify fibronectin as a promising target capable of disrupting the self‐perpetuating ECM‐myofibroblast loop to mitigate fibrosis progression.

## 2 Results

### 2.1 Increased Fibronectin Forms the Structural Backbone of Fibrotic Intestinal ECM

**FIGURE 1:**
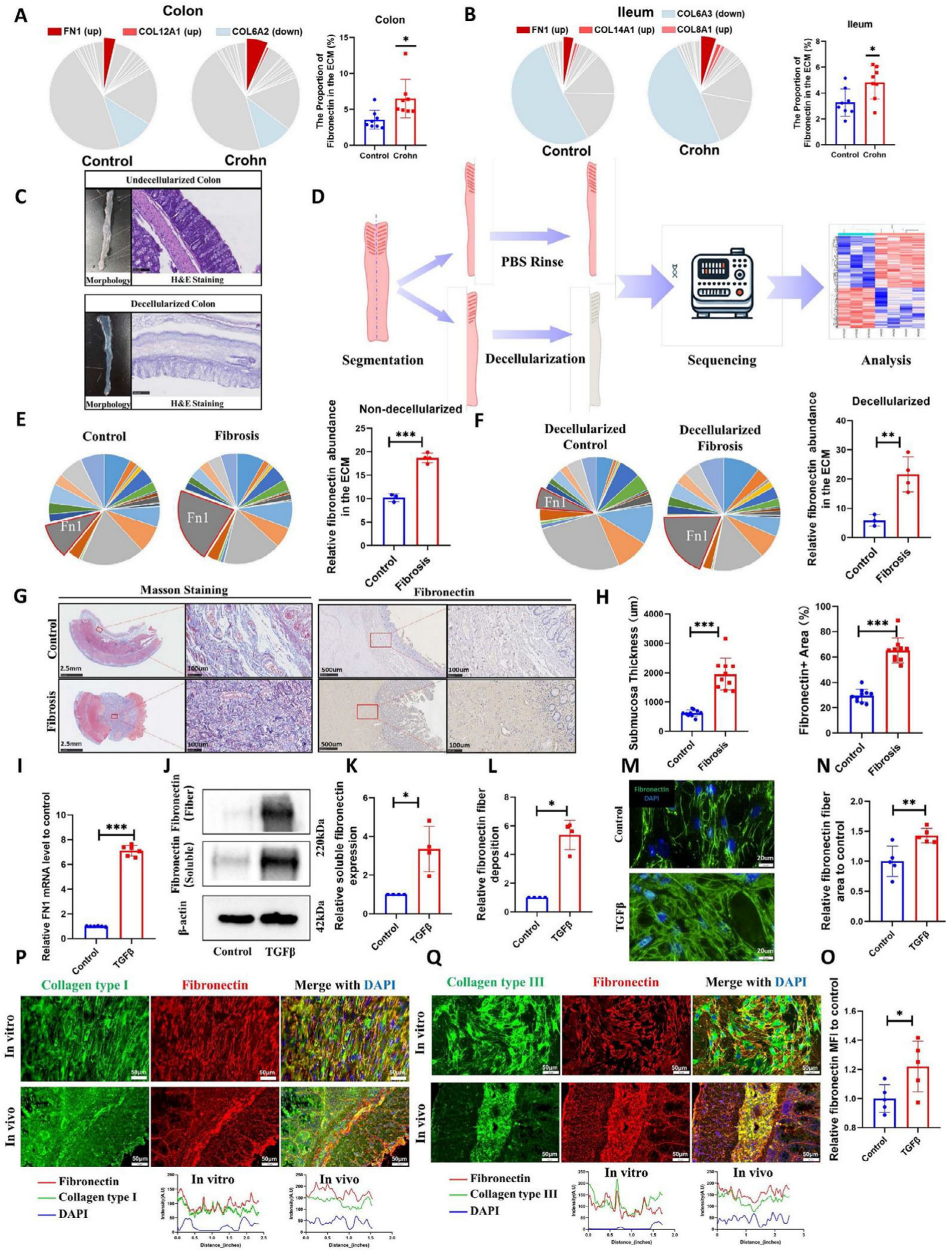
Fibronectin, as the ECM scaffold, accumulates in fibrotic intestines. (A, B) Matrixome analysis in clinical specimens (A colon and B ileum). In the pie chart, red refers to increased ECM in Crohn's intestine; blue refers to decreased ECM in Crohn's intestine. The bar plots show the relative abundance of fibronectin in the control and Crohn's group (*n* = 8). (C) Gross specimens and H&E staining of native and decellularized intestinal tissues. (D) Schematic workflow of intestinal decellularization and proteomic sequencing. Mice colons were bisected longitudinally; one segment underwent PBS rinsing (control), and the other underwent decellularization. Both were subjected to proteomic sequencing and analysis. Groups: control (*n* = 3), fibrosis (*n* = 4). (E, F) Matrixome analysis in the intestinal fibrosis model. The gray region in the pie chart refers to fibronectin. The bar plots show the relative abundance of fibronectin in the control and fibrosis group under native or decellularized conditions. (G) Representative Masson's trichrome staining and fibronectin IHC of healthy (Control group, *n* = 10) and fibrotic (Fibrosis group, *n* = 10) human intestinal tissues. (H) Quantification of submucosal thickness and fibronectin abundance in human specimens. (I) Fibronectin transcription levels change in fibrotic cell models (*n* = 6). (J–L) Relative abundance of soluble fibronectin and fibronectin fibrils in fibrotic cell models (*n* = 3). (M–O) Differences in fibronectin abundance and morphological changes in cell models (*n* = 5). (P, Q) Colocalization of fibronectin fibrils with type I/III collagen fibers in vitro and in vivo. Human intestinal fibroblasts CCD18Co were used for cell models. Data are presented as mean ± SD. ^*^ *p* < 0.05; ^**^ *p* < 0.01; ^***^ *p* < 0.001. Continuous data were analyzed using Student's t‐test. Nonparametric distributions were analyzed by Mann‐Whitney/Wilcoxon rank‐sum tests. IHC, immunohistochemistry; ECM, extracellular matrix; DSS, dextran sulfate sodium salt; TNBS, 2,4,6‐trinitrobenzenesulfonic acid. MFI, mean fluorescence intensity. Scale bar: 2.5 mm and 100 µm (G, Masson staining); 500 and 100 µm (G, Fibronectin); 20 µm (M); 100 µm (P and Q).

To identify key ECM proteins in intestinal fibrosis, we first performed matrisomic analysis on ileal and colonic tissues from Crohn's disease patients [18], quantifying the composition of major ECM proteins. Results demonstrated that fibronectin was the only protein significantly enriched in fibrotic ileum and colon (Figure 1A,B). Subsequently, we established a dextran sulfate sodium salt (DSS)‐induced intestinal fibrosis model. Decellularized intestines were generated to eliminate the confounding effects of intracellular proteins (Figure 1C,D). Notably, fibronectin enrichment persisted in both decellularized and native fibrotic intestine tissues, showing striking consistency with clinical matrisomics (Figure 1E,F). This established fibronectin as a hallmark of fibrotic ECM.

To further delineate fibronectin's expression and distribution in intestinal fibrosis, we collected clinical specimens and established two mouse fibrosis models: DSS‐induced (innate immune‐driven, ulcerative colitis‐like) and 2,4,6‐trinitrobenzenesulfonic acid (TNBS)‐induced (adaptive immune‐driven, Crohn's disease‐like) [19]. Histological staining (HE, Masson's trichrome, Sirius red) revealed significant submucosal thickening in fibrotic intestines, with excessive ECM deposition predominantly localized to the submucosa (Figure S1A–C). Immunohistochemistry confirmed markedly elevated fibronectin expression primarily within the submucosal layer, aligning with pathological ECM distribution (Figure 1G,H; Figure S1D–G).

To evaluate native ECM fibrillogenesis and pathological deposition in vitro, we employed human intestinal fibroblasts (HIFs) CCD18Co to generate a reductionist intestinal fibrosis model [3]. In previous studies, fibronectin was usually extracted without distinguishing insoluble fibronectin fibril from soluble fibronectin. To address this oversight, we separated soluble fibronectin fibrils from insoluble fibronectin fibrils using deoxycholic acid salts [20], and then measured their abundance in parallel. Immunofluorescence further distinguished ECM fibrils from intracellular soluble fibronectin by spatial and morphological features. Fibroblast activation significantly increased fibronectin transcription (Figure 1I), elevating both intracellular soluble fibronectin and insoluble ECM fibrils (Figure 1J–L), and promoted ECM fibril densification and thickening (Figure 1M–O).

Subsequent spatial mapping of fibronectin in the ECM revealed its critical colocalization with core intestinal ECM components (collagen I/III) across in vivo and in vitro models (Figure 1P,Q). We further investigated fibronectin and ECM colocalization following intracellular proteins eliminated. After decellularization, immunofluorescence co‐staining of intestinal sections showed that fibronectin and collagen signals in the mucosal layer were substantially diminished, whereas strong co‐localization of fibronectin with collagen persisted in the submucosa (Figure S1H,I). These findings implicate fibronectin as a structural ECM scaffold whose removal may destabilize deposition and potentially ameliorate intestinal fibrosis.

### 2.2 Fibronectin Depletion Ameliorates Intestinal Fibrosis

**FIGURE 2:**
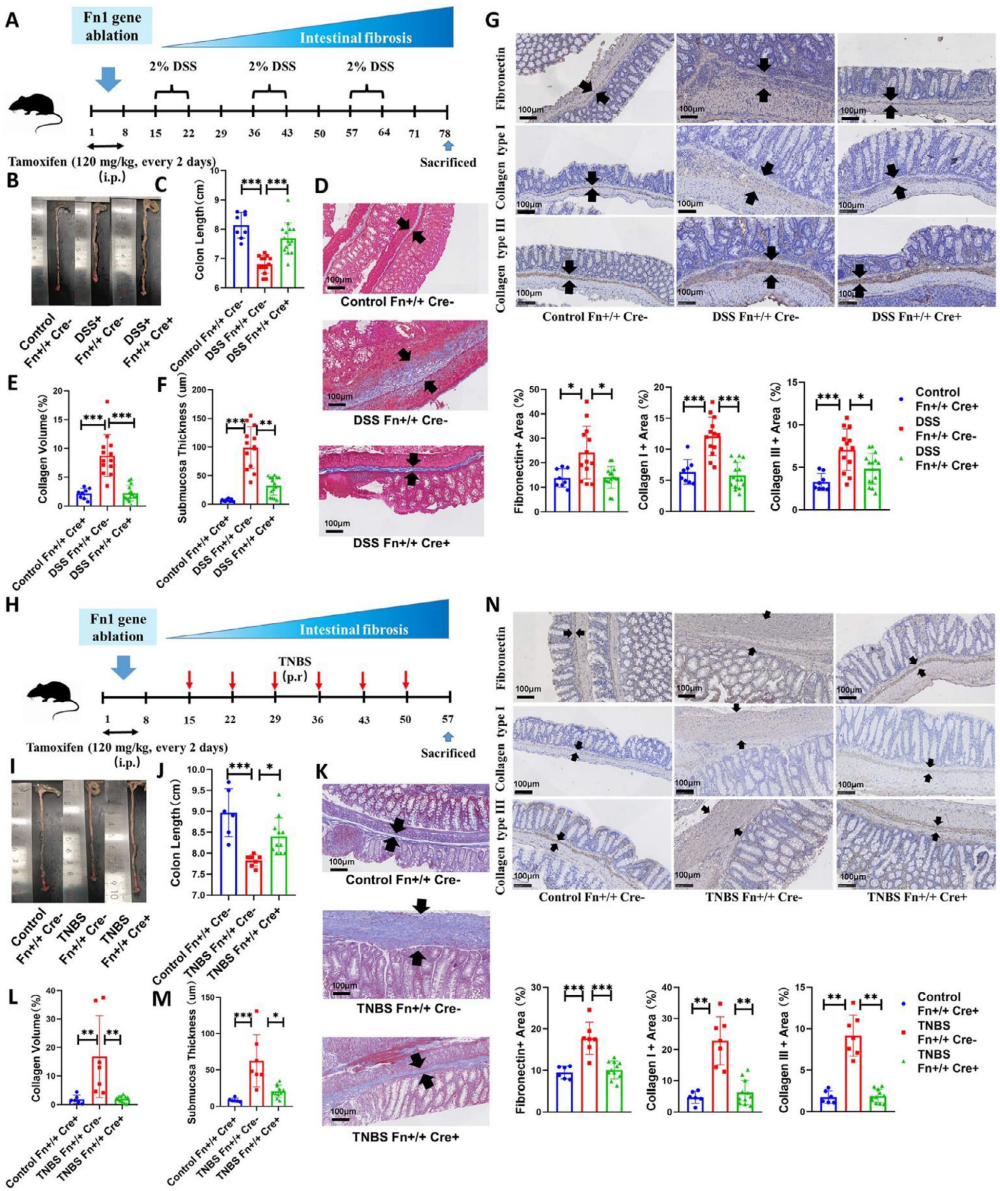
Fibronectin genetic ablation ameliorates intestinal fibrosis. (A) Workflow for DSS model in knockout mice. Mice (6–8 weeks) received tamoxifen (120 mg/kg every 2 days, i.p.) for one week, rested one week, then underwent three cycles of 2% DSS in drinking water (1 week) followed by regular water (2 weeks). Tissues were harvested after all cycles were completed. Groups: Control Fn+/+ Cre‐ (*n* = 8), DSS Fn+/+ Cre‐ (*n* = 13), DSS Fn+/+ Cre+ (*n* = 15). (B, C) Intestinal length differences in the DSS model. (D–F) Representative Masson's trichrome staining, collagen volume fraction, and submucosal thickness quantification in the DSS model. (G) Representative IHC staining and quantification of fibronectin, collagen I, and collagen III in the DSS model. (H) Workflow for TNBS model in knockout mice. Mice received tamoxifen as in (A), rested one week, then underwent weekly intrarectal TNBS administration (0.1 mL, increasing concentration. The detail could be found in Section S2) for 6 weeks. Tissues were harvested after all cycles were completed. Groups: Control Fn+/+ Cre‐ (*n* = 6), TNBS Fn+/+ Cre‐ (*n* = 7), TNBS Fn+/+ Cre+ (*n* = 11). (I–M) Intestinal length differences, representative Masson's trichrome staining, collagen volume fraction, and submucosal thickness quantification in the TNBS model. (N) Representative IHC staining and quantification of fibronectin, collagen I, and collagen III in the TNBS model. Data are presented as mean ±SD. ^*^ *p* < 0.05; ^**^ *p* < 0.01; ^***^ *p* < 0.001. Continuous data were analyzed using ANOVA for unpaired groups. Nonparametric distributions were analyzed by Kruskal‐Wallis tests, with Dunn's post hoc testing for multiple comparisons. IHC, immunohistochemistry; ECM, extracellular matrix; DSS, dextran sulfate sodium salt; TNBS, 2,4,6‐trinitrobenzenesulfonic acid. Scale bar: 100 µm.

To validate this hypothesis, we established a fibronectin‐depleted mouse model. Because complete fibronectin knockout causes embryonic lethality and fibroblasts are the primary producers and assemblers of fibronectin, we generated fibroblast‐specific Fn1 knockout mice [7, 21, 22]. At baseline, Fn1‐depleted mice showed no significant differences in gross phenotypes compared to controls (Figure S2A–D). After knockout, Fn1 transcription was markedly reduced, accompanied by significant downregulation of major collagen transcripts; however, submucosal fibronectin‐collagen ECM remained comparable (Figure S2E,F). The discrepancy between transcriptional and staining results may be due to the relatively stable state of mature ECM in healthy intestines, where reduced transcription is insufficient to noticeably decrease deposited ECM. Using this model, we induced intestinal fibrosis via DSS and TNBS challenges and quantified fibrosis parameters. In DSS‐treated mice, Fn1 depletion increased colon length, reduced collagen volume fraction and submucosal thickness, and attenuated deposition of fibronectin, collagen I, and collagen III. Fn1 knockout significantly reversed these pathological changes (Figure 2A–G). Subsequently, TNBS induction robustly elevated intestinal fibrotic scoring, while Fn1 knockout substantially ameliorated these manifestations (Figure 2H–N).

### 2.3 Fibronectin Depletion Transforms Primary Fibrotic Intestinal Wall Thickening into Primary Inflammatory Thickening

**FIGURE 3:**
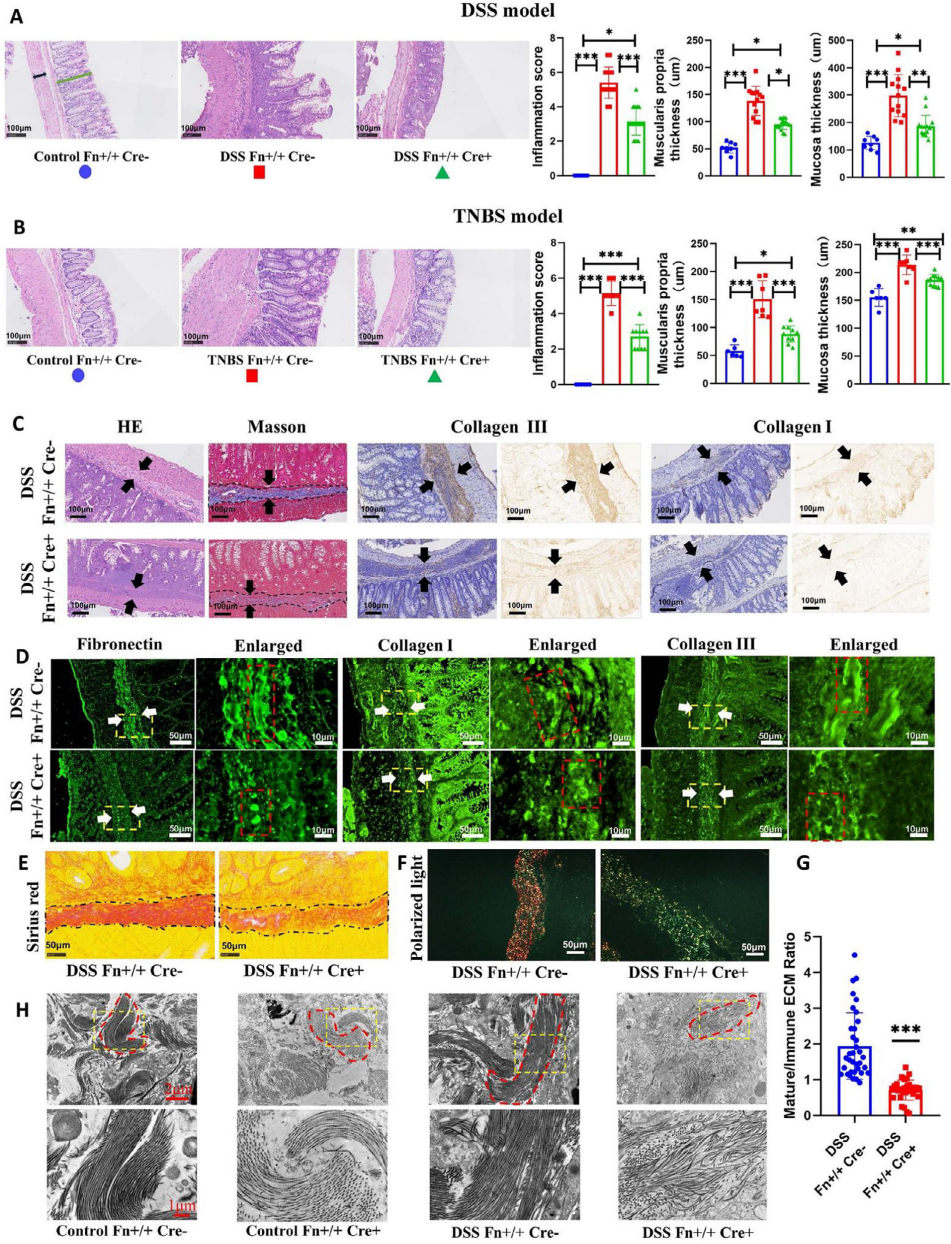
Fibronectin deficiency alters extracellular matrix fiber morphology. (A) Representative H&E staining and evaluation of inflammation score, mucosal layer thickness, and muscularis propria thickness in the DSS model. Groups: Control Fn+/+ Cre‐ (*n* = 8), DSS Fn+/+ Cre‐ (*n* = 13), DSS Fn+/+ Cre+ (*n* = 15). Black arrows indicate representative measurement sites for the muscularis propria, and green arrows indicate those for the mucosal layer. Detailed criteria for inflammation scoring are provided in the Supporting Information. (B) Representative H&E staining and parameter evaluation in the TNBS model. Groups: Control Fn+/+ Cre‐ (*n* = 6), TNBS Fn+/+ Cre‐ (*n* = 7), TNBS Fn+/+ Cre+ (*n* = 11). The measurement and scoring criteria are the same as those in the DSS model. (C) Morphological differences in main ECM components between knockout and wild‐type mice at matched submucosal thickness. At equivalent thickness, DSS/TNBS‐exposed wild‐type submucosa exhibited fibrosis‐driven thickening with abundant ECM deposition, while Fn1‐knockout submucosa showed inflammation‐driven thickening predominated by infiltrating cells with minimal ECM accumulation. (D) Immunofluorescence reveals ECM protein morphology at matched submucosal thickness (bracketed by white arrows; yellow boxes: magnified regions). Wild‐type tissues displayed mature, wavy, coarse fibers of fibronectin, collagen I, and collagen III. Fn1 knockout resulted in immature punctate, non‐fibrous deposits (red boxes: representative single‐fiber morphology). (E) Representative Sirius Red staining image. (F) Representative Sirius Red staining image under polarized light. (G) Statistical analysis of the mature/immature ECM ratio. (DSS Fn+/+ Cre‐ group: 35 sights from 13 individual; DSS Fn+/+ Cre+ group: 32 sights from 15 individual). (H) Ultrastructure of submucosal collagen fibers (TEM). Healthy mice (representative image from ≥15 fields/3 mice) had short collagen bundles comprising thick, dense fibers. Fn1 knockout (≥15 fields/3 mice) showed notably thinner bundles. Fibrotic conditions (≥20 fields/4 mice) exhibited thicker, denser, and elongated collagen fibers. Fn1 knockout under fibrosis (≥20 fields/4 mice) yielded sparse, broom‐distributed collagen fibrils, suggesting impaired fibrillogenesis (yellow boxes: magnified regions; red boxes: single‐fiber morphology). Data are presented as mean ±SD. ^*^ *p* < 0.05; ^**^ *p* < 0.01; ^***^ *p* < 0.001. Continuous data were analyzed using Student's t‐test or ANOVA for unpaired groups. Nonparametric distributions were analyzed by Kruskal‐Wallis tests, with Dunn's post hoc testing for multiple comparisons. IHC, immunohistochemistry; ECM, extracellular matrix; DSS, dextran sulfate sodium salt; TNBS, 2,4,6‐trinitrobenzenesulfonic acid; TEM, transmission electron microscope. Scale bar: 100 µm (A–C); 50 µm (D) and (D, enlarged); 2 µm (E) and 1 µm (Enlarged).

Beyond quantitative analysis of submucosal thickness and collagen volume fraction, we further evaluated intestinal inflammation scoring, mucosal layer thickness, and muscular layer thickness to better understand fibrotic intestinal tissue reconstruction mediated by direct targeting of ECM protein. In DSS‐induced fibrotic intestines, inflammatory scores were significantly higher than in controls. While fibronectin knockout markedly reduced inflammation scores, they remained significantly elevated relative to healthy controls. Both mucosal and muscularis propria layers exhibited significant thickening in fibrotic intestines; Fn1 depletion substantially reduced their thickness, yet they remained significantly thicker compared to controls (Figure 3A). In the TNBS model, fibronectin knockout similarly attenuated TNBS‐induced inflammation, mucosal thickening, and muscularis propria thickening, though significant differences persisted versus healthy controls (Figure 3B).

Intriguingly, Fn1 ablation conferred benefits beyond overall amelioration of intestinal fibrosis. In Fn1‐knockout individuals, although the submucosal thickness of those severely impaired segments showed no significant difference versus DSS‐induced Cre‐negative controls, collagen components were remarkably depleted, and the submucosa predominantly exhibited inflammatory cell infiltration rather than ECM deposition (Figure 3C). This suggests that even with comparable submucosal thickening, Fn1 knockout manifests as reversible inflammatory expansion rather than irreversible fibrotic thickening.

Immunofluorescence revealed distinct matrix architectures: fibrosis‐associated fibronectin, collagen I, and collagen III formed mature, coarse fibrous structures, whereas Fn1 ablation transformed these matrices into immature, sparse non‐fibrous assemblies. (Figure 3D). We subsequently performed Sirius Red staining. When comparing regions with similar submucosal thickness, the intestinal ECM in Fn1‐knockout mice was significantly sparser (Figure 3E). Under polarized light, Sirius Red‐stained tissues display mature collagen fibers in orange‐red and immature collagen fibers in yellow‐green. We examined submucosal areas of comparable thickness and observed that fibrotic submucosa contained abundant mature collagen fibers, predominantly orange‐red. Following Fn1 knockout, mature collagen fibers in the submucosa were significantly reduced, with the staining shifting to predominantly yellow‐green (Figure 3F). We then quantified the ratio of mature to immature collagen in the submucosa and found that this ratio was significantly lower after Fn1 ablation (Figure 3G).

Ultrastructurally, control submucosa displayed tightly packed, dense collagen fibril bundles. Fn1‐knockout specimens demonstrated significantly reduced fibril density. Fibrosis induction exacerbated the coarse, densely packed fibril arrangement, whereas Fn1 knockout induced disorganized, broom‐shaped fibril dispersion (Figure 3H). These findings indicate that the anti‐fibrotic effect of fibronectin deficiency is mediated primarily by regulating ECM accumulation and remodeling, rather than by modulating inflammation.

### 2.4 Fibronectin as the Structural Scaffold Directly Governs ECM Assembly

**FIGURE 4:**
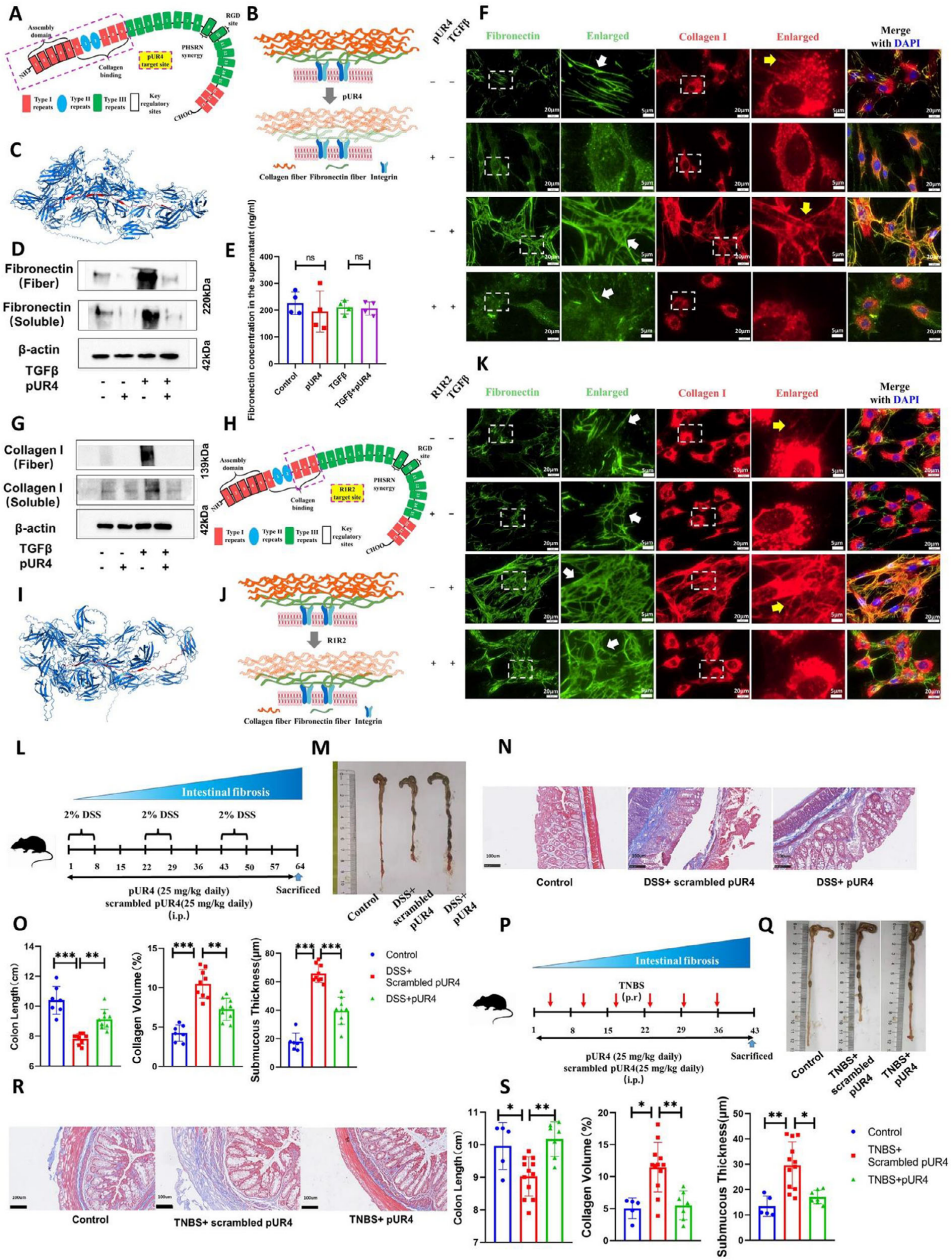
Blocking fibronectin assembly impedes fibrillogenesis and ameliorates intestinal fibrosis. (A) Schematic of pUR4 targeting fibronectin domains (detailed in Supporting Information). (B) Proposed mechanism of pUR4 action on extracellular matrix. (C) Molecular docking of pUR4 with fibronectin. (D) The relative abundance of soluble fibronectin and fibronectin fibers with pUR4 treatment. (E) ELISA of fibronectin in cell culture supernatants. (F) Representative images (of three biological replicates) depicting pUR4 effect on fibronectin (white arrows) and collagen (yellow arrows) fibers; white boxes show magnified regions. (G) The relative abundance of soluble collagen and collagen fibers with pUR4 treatment. (H) Schematic of R1R2 targeting fibronectin domains. (I) Molecular docking of R1R2 with fibronectin. (J) Proposed mechanism of R1R2 action on ECM. (K) R1R2 effects on fibronectin/collagen fibers; conventions as in (F). (L) DSS model dosing protocol: Mice received daily i.p. pUR4 (25 mg/kg) or scrambled pUR4 (equimolar) from DSS initiation until endpoint (Control, *n* = 7; DSS+scrambled pUR4, *n* = 9; DSS+pUR4, *n* = 9). (M, N) Macroscopic colon images (M) and Masson's trichrome staining (N) in the DSS model. (O) Quantification of colon length, collagen volume fraction, and submucosal thickness. (P) TNBS model dosing: Protocol as in (L) with TNBS challenge (Control, *n* = 5; TNBS+scrambled pUR4, *n* = 7; TNBS+pUR4, *n* = 12). (Q, R) Macroscopic colon images (M) and Masson's trichrome staining (N) in the TNBS model. (S) Quantification of colon length, collagen volume fraction, and submucosal thickness. Human intestinal fibroblasts CCD18Co were used for in vitro studies. Data are presented as mean ±SD. ^*^ *p* < 0.05; ^**^ *p* < 0.01; ^***^ *p* < 0.001. Continuous data were analyzed using ANOVA for unpaired groups. Nonparametric distributions were analyzed by Kruskal‐Wallis tests, with Dunn's post hoc testing for multiple comparisons. IHC, immunohistochemistry; ECM, extracellular matrix; DSS, dextran sulfate sodium salt; TNBS, 2,4,6‐trinitrobenzenesulfonic acid. Scale bar: 20 µm (J. K) and 5 µm (Enlarged); 100 µm (N, R).

To investigate how fibronectin inhibition results in the collapse of the whole fibrotic tissue, we examined alterations in major ECM fibrous components through targeted inhibition of fibronectin fibrillogenesis in vitro. The short peptide pUR4, derived from Streptococcus pyogenes surface proteins, was confirmed to bind the N‐terminal type I domain of fibronectin via molecular docking studies, with molecular dynamics simulations demonstrating high‐affinity interactions that effectively block fibronectin assembly (Figure 4A–C; Figure S3B) [23]. pUR4 treatment substantially reversed cellular activation‐induced increases in intracellular soluble fibronectin expression and ECM fibronectin fibril deposition, as well as collagen I (Figure 4D–G). However, secreted fibronectin levels in cell supernatants remained completely unaffected (Figure 4E). In unactivated HIFs, sparse fibronectin fibrils constitutively form delicate filamentous networks. pUR4 treatment completely abolished these fibrillar structures, leaving only intracellular unassembled fibronectin. Activated HIFs generated dense, coarse fibronectin fibrils. Following pUR4 intervention, these pathologically thickened fibers were virtually eradicated, with only residual immature, shortened filamentous fragments remaining (Figure 4F).

In unactivated HIFs, collagen predominantly resides intracellularly as soluble protein pools with minimal filamentous fiber formation. Upon cellular activation, HIFs synthesize abundant dense and compacted collagen fibers. Crucially, blocking fibronectin assembly concurrently eliminated collagen fibrillogenesis (Figure 4F), suggesting collagen fiber formation may depend on fibronectin scaffolding. To test this, we employed the Streptococcus equi‐derived peptide R1R2, which targets the interface between fibronectin type I domains 8–9 and collagen [24]. Molecular docking precisely mapped its binding site to these domains, while molecular dynamics simulations confirmed high‐affinity interactions that disrupt fibronectin‐collagen binding (Figure 4H–J; Figure S3C). When administered to unactivated HIFs, R1R2 eliminated all filamentous collagen fibers without altering fibronectin fibril morphology. In activated HIFs, identical R1R2 treatment abolished dense collagen matrices while preserving native fibronectin networks, leaving only intracellular soluble collagen reservoirs intact (Figure 4K).

### 2.5 Blocking Fibronectin‐Fibronectin Binding, but not Fibronectin‐Collagen Binding, Ameliorates Intestinal Fibrosis

**FIGURE 5:**
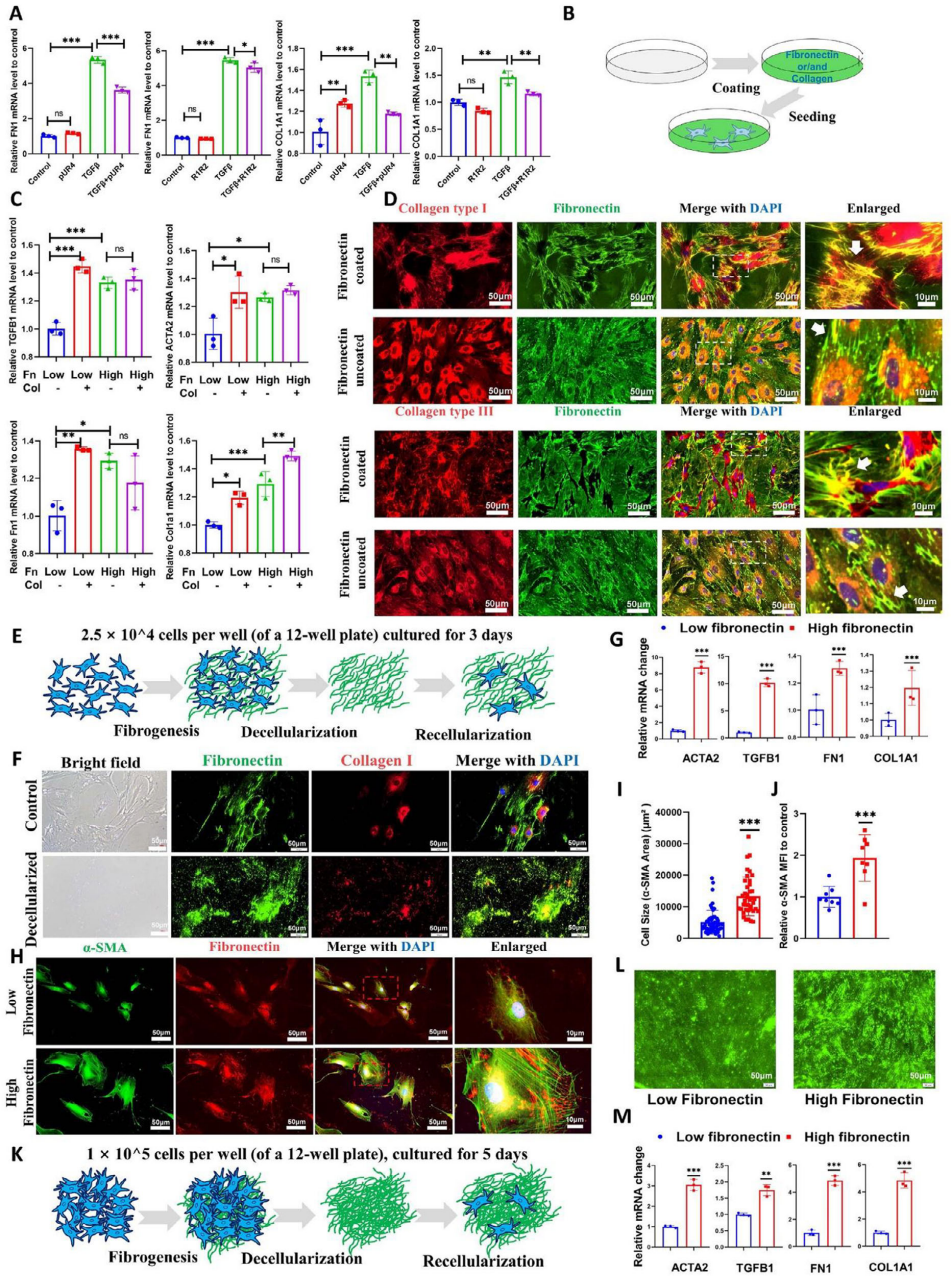
Fibronectin in the extracellular matrix regulates cellular activation and fibrillogenesis. (A) Transcriptional impact of pUR4 and R1R2 on FN1 and COL1A1. (B) Schematic workflow for ECM coating experiments. (C) The gene expression changes of fibroblasts in different ECM coating matrices. low FN (5µg/mL), high FN (50µg/mL), and collagen (5µg/mL). (D) Collagen I/III morphology on FN‐coated vs. non‐coated matrices. Background FN fluorescence was high on FN‐coated matrices with low intracellular signal, but low in non‐coated systems with prominent intracellular FN accumulation (yellow circles: single cells; white boxes: magnified regions; white arrows: ECM fibers). (E) Decellularization model workflow. HIFs were cultured at 2.5 × 10^4 cells per well (12‐well plate) for 3 days before decellularized. Low fibronectin group: derived from CCD18co. High fibronectin group: derived from TGFβ‐treated CCD18co. (F) Validation of decellularization: Pre‐treatment showed intact cell structures, fibrous FN, and soluble collagen. Post‐decellularization preserved FN fibers while eliminating cells and collagen. (G) Transcriptional differences induced by varying FN levels in decellularized matrices. (H) Cell morphology modulation by FN density: Low FN promoted spindle shapes with diffuse α‐SMA; high FN induced flattened, polygonal cells with enlarged size and α‐SMA‐rich stress fibers. (I, J) Quantification of cell size and α‐SMA MFI (8 sights from 4 biological replicates). (K) Thick‐layer decellularization model workflow. HIFs were cultured at 1 × 10^5 cells per well (12‐well plate) for 5 days before decellularized. Low fibronectin group: derived from CCD18co. High fibronectin group: derived from TGFβ‐treated CCD18co. (L) Presentation of the remaining fibronectin ECM in the thick‐layer decellularization model. In the low fibronectin group, fibronectin is diffusely distributed across the dish bottom, appearing as punctate spots along with a small number of thin filaments. In the high fibronectin group, a greater amount of fibronectin is diffusely distributed across the entire dish bottom, accompanied by abundant thick, fibrous bundles. (M) Transcriptional differences in the thick‐layer decellularization model. Data are presented as mean ±SD. ^*^ *p* < 0.05; ^**^ *p* < 0.01; ^***^ *p* < 0.001. Continuous data were analyzed using Student's t‐test or ANOVA for unpaired groups. Nonparametric distributions were analyzed by Mann‐Whitney/Wilcoxon rank‐sum tests or Kruskal‐Wallis tests, with Dunn's post hoc testing for multiple comparisons. FN, fibronectin; ECM, extracellular matrix; MFI, mean fluorescence intensity. Scale bar: 50 and 10 µm (Enlarged).

To validate the therapeutic potential of inhibiting fibronectin assembly in intestinal fibrosis, pUR4 demonstrated robust protective effects in DSS‐induced models. Administration of pUR4 significantly attenuated key fibrotic indicators—including colon shortening, collagen deposition area, and submucosal thickening—compared to scrambled peptide controls (Figure 5L–O). Parallel protection was replicated in TNBS models (Figure 5P–S). The results of transcriptomics also showed that after receiving pUR4 treatment, the transcription levels of ECM‐related genes in the intestine were significantly reduced (Figure S8). Contrastingly, R1R2‐mediated suppression of collagen fibrillogenesis minimally impacted fibrosis progression in vivo (Figure S6A–F), despite complete disruption of collagen fibers. These findings establish fibronectin fibrillogenesis as the primary initiating scaffold for ECM formation, suggesting post‐assembly interventions targeting downstream ECM components may exhibit limited efficacy due to the temporal dependency of matrix orchestration.

### 2.6 Fibronectin Matrix Structure Dictates Intestinal Fibroblast Activation State

Mechanistically, both pUR4 and R1R2 specifically disrupt fibronectin fibrillogenesis without inhibiting fibronectin/collagen transcription or translation [23, 24]. Paradoxically, our data revealed that TGFβ‐activated HIFs exhibited significant downregulation in both mRNA expression and de novo synthesis of fibronectin and collagen following pUR4 or R1R2 interventions (Figure 5A). Since ECM deposition was markedly reduced post‐treatment and the ECM provides critical mechanotransduction cues, we propose that the resulting fibronectin/collagen‐deficient ECM fails to sustain the mechanical signaling required for persistent fibroblast activation. This aligns with established paradigms in which ECM stiffness perpetuates fibroblast activation. Thus, we hypothesize that the collapse of the tension‐sensing ECM architecture initiates a feedback suppression of matrix biosynthesis pathways, ultimately suppressing fibroblast‐driven matrix production.

To validate this hypothesis, we established ECM‐coated matrices simulating extracellular matrices with varying fibronectin and/or collagen densities (Figure 5B). Compared to HIFs cultured on low‐density fibronectin/collagen‐coated matrices, HIFs grown on high‐density coatings demonstrated significantly increased expression of activation markers, including fibronectin and collagen themselves (Figure 5C). Collagen coating can boost the cellular activation levels under low fibronectin coating conditions, but not under high fibronectin coating conditions, probably indicating saturated mechano‐activated effects. This saturation effect likely stems from dual constraints: a biomechanical equilibrium and competitive occupation of cellular adhesion sites. On the one hand, high‐density fibronectin may establish a mechanical equilibrium with cells; supplementing collagen does not alter this balanced state to provide additional mechanical force [12]. On the other hand, fibronectin's stronger effective affinity allows it to dominantly occupy adhesion sites when abundant, thereby marginalizing collagen's contribution and limiting further activation [25].

We then assessed fibrillar morphology in HIFs via immunofluorescence under varying fibronectin coatings. Fibronectin fibril organization showed no significant difference between uncoated and high‐density fibronectin‐coated matrices (though fibrils appeared slightly sparser in uncoated samples). HIFs on uncoated matrices exhibited primarily intracellular collagen accumulation, whereas fibronectin‐coated matrices markedly enhanced fibrillogenesis, with collagen adopting organized fibrous structures. Notably, collagen fiber formation displayed directional specificity: fibrils exclusively aligned along pre‐existing dense collagen tracks and failed to extend over uniformly fibronectin‐coated areas. This indicates that a fibronectin‐rich environment promotes dense collagen fiber assembly primarily on preexisting fibronectin fibers (Figure 5D).

To better mimic fibronectin fibril‐mediated effects in native extracellular matrices, we developed a fibroblast‐specific decellularization protocol (Figure 5E). Post‐decellularization, cellular structures were fully eliminated with near‐complete depletion of soluble components, retaining only insoluble ECM fibrils predominantly composed of fibronectin (Figure 5F). Using decellularized matrices from activated and non‐activated HIFs—yielding dense versus loose fibronectin fibril architectures—we reseeded fresh HIFs onto these scaffolds. Cells on dense fibronectin fibrils exhibited significantly upregulated transcription of activation‐associated genes (Figure 5G), concurrent with elevated αSMA expression forming distinct stress fibers and a marked increase in cell volume (Figure 5H–J). However, although fibronectin exhibits high adsorption affinity to cells [25], we could not guarantee that all reseeded cells would interact with the decellularized matrix. Therefore, we increased the number of ECM‐producing cells and extended the duration of ECM deposition to ensure complete coverage of the culture dish bottom (Figure 5K,L), thereby guaranteeing that cells would inevitably interact with the decellularized matrix. The impact of such thick‐layered and subsequently decellularized ECM on the activation level of reseeded cells was consistent with previous experimental results (Figure 5M), demonstrating the robustness of this model. These results collectively establish that elevated ECM fibronectin content promotes HIF activation, while fibronectin depletion substantially reduces activation.

### 2.7 Fibronectin Anchoring to Intestinal Fibroblasts via Integrin α5β1

**FIGURE 6:**
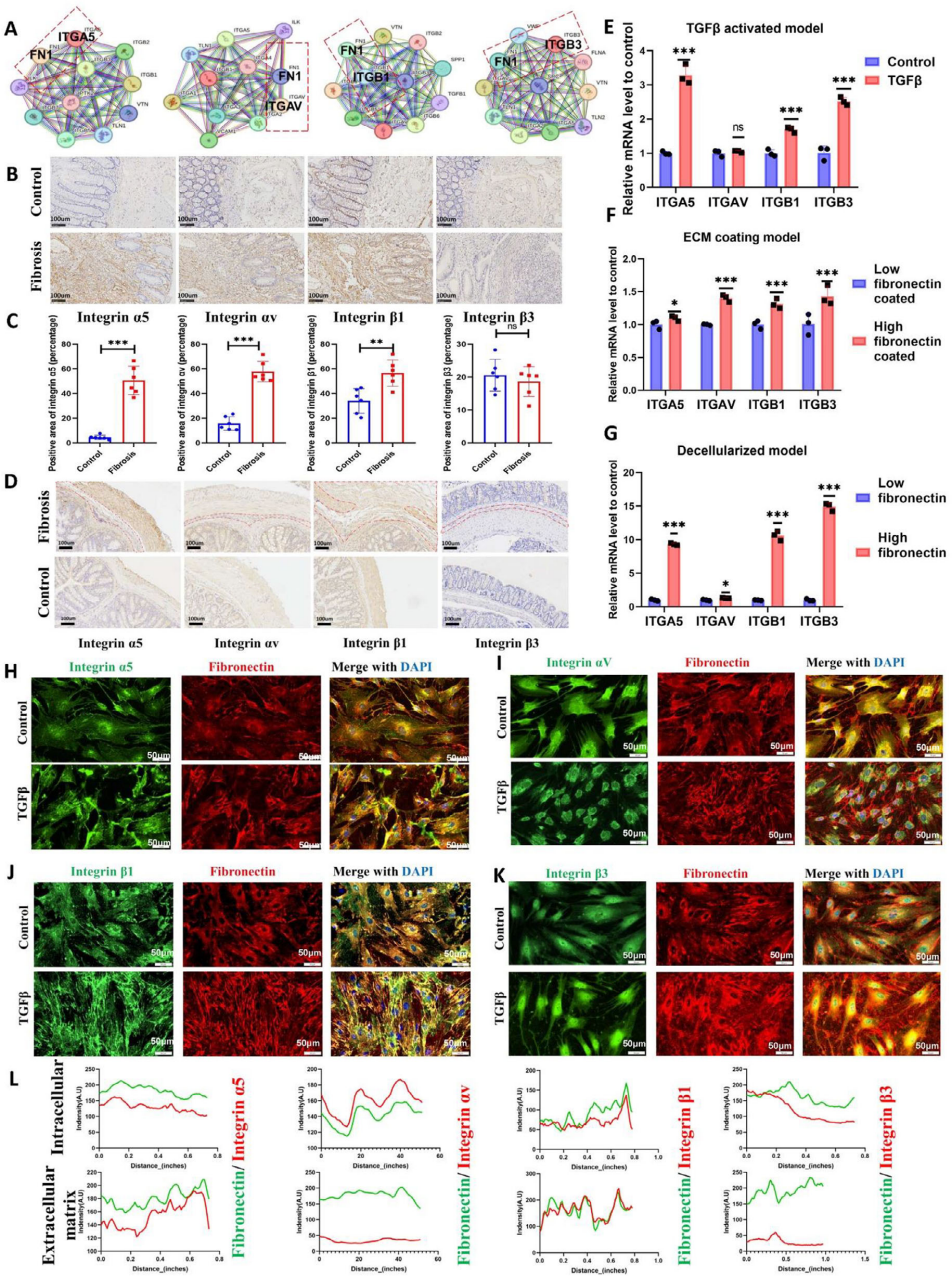
Integrin α5β1 serves as the cellular receptor bridging ECM fibronectin to the cell. (A) STRING protein interaction map of major fibronectin integrin receptors. (B, C) Representative images and quantification of major integrin receptor expression in clinical specimens (Control, *n* = 6; Fibrosis, *n* = 6). (D) Representative images of major integrin receptors in animal models. (E) Integrin expression differences in fibroblasts under TGFβ activation (*n* = 3). (F) Integrin expression differences in fibroblasts in the ECM coating model (*n* = 3). (G) Integrin expression differences in fibroblasts in the decellularization model (*n* = 3). (H–K) Distribution of integrins and fibronectin in fibroblasts and their pericellular ECM. (L) Co‐localization analysis of integrins and fibronectin. The selected parts for co‐localization analysis can be found in Figure S7. Human intestinal fibroblasts CCD18Co were used for cell models. Data are presented as mean ±SD. Continuous data were analyzed using Student's t‐test. Nonparametric distributions were analyzed by Mann‐Whitney. ^*^ *p* < 0.05; ^**^ *p* < 0.01; ^***^ *p* < 0.001. FN, fibronectin; ECM, extracellular matrix. Scale bar: 100 µm (B, D); 50 µm (H–K).

Extracellular matrices transmit mechanical signals to cells via surface receptors, thereby modulating cellular activation states. Integrins—transmembrane heterodimers composed of α and β subunits—serve as primary mediators of ECM‐to‐cell signaling [26]. Key fibronectin receptors include integrins α5β1, αvβ1, and αvβ3, whereas integrin α4β1 (primarily expressed in hematopoietic cells) was excluded [27]. STRING protein interaction analysis confirmed direct binding between fibronectin and subunits α5, αv, β1, and β3 (Figure 6A), which was subsequently validated in human clinical specimens. In fibrotic human intestinal tissues, integrins α5, αv, and β1 showed significantly upregulated expression in the submucosa relative to healthy controls, while β3 remained minimally expressed in both states (Figure 6B,C). Mice studies revealed strong integrin positivity in the muscularis propria, obscuring submucosal expression analysis; however, prominent upregulation of α5, αv, and β1 was distinctly observed in fibrotic mouse colon submucosa, with β3 consistently demonstrating low expression across all conditions (Figure 6D).

To identify specific integrins mediating fibronectin‐induced effects on HIFs, we analyzed activated HIFs and observed markedly upregulated expression of integrin subunits α5, β1, and β3, while αv remained unchanged (Figure 6E). Elevated fibronectin exposure in both fibronectin‐coated cell culture models and decellularized matrices with dense fibronectin remnants universally induced upregulation of all evaluated integrins (Figure 6F,G). Although α5 and β1 demonstrated the most stable overexpression under fibrotic conditions and fibronectin stimulation, we could not conclusively establish α5β1 integrin as the dominant mediator in HIFs at this stage.

Colocalization analysis ultimately provided definitive evidence. Paired immunofluorescence staining of integrins and fibronectin in activated versus non‐activated HIFs revealed persistently high spatial coupling between fibronectin fibrils and integrins α5/β1 regardless of activation status or fibronectin abundance. Both exhibited co‐distributed fibrillar architecture, whereas αv and β3 demonstrated diffuse intracellular localization (Figure 6H–K). To more clearly delineate the spatial relationship between the integrins and fibronectin, we performed separate colocalization analyses for the cellular and extracellular matrix compartments. The results showed that all four integrins exhibited high colocalization with fibronectin within the cells. However, in the extracellular matrix, only integrins α5 and β1 maintained a strong colocalization relationship with fibronectin (Figure 6L). This confirms that integrin α5β1 exclusively mediates fibronectin‐HIF connectivity in intestinal fibrosis.

### 2.8 Fibronectin Triggers Mechanotransduction Pathways of Intestinal Fibroblasts through Integrin α5β1

**FIGURE 7:**
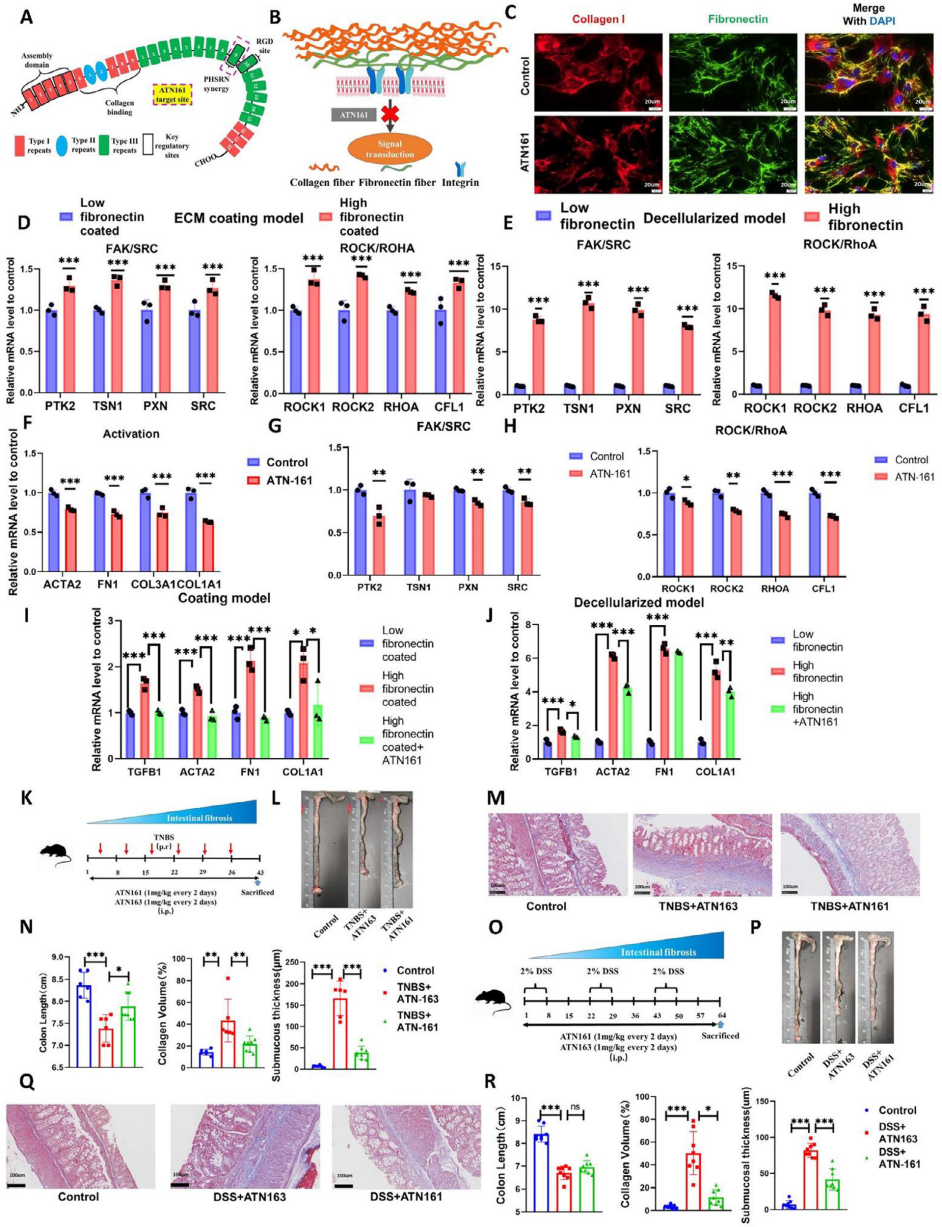
Fibronectin regulates intestinal fibrosis via integrin‐mediated mechanotransduction. (A, B) Schematic representations of ATN161 acting on fibronectin domains. (C) ATN161 does not alter ECM fibronectin or collagen morphology. (D) Gene expression differences in integrin‐mediated mechanotransduction pathways (FAK/SRC and ROCK/RhoA) in the ECM coating model. (E) Gene expression differences in integrin‐mediated mechanotransduction pathways in the decellularization model. (F–H) Impact of ATN161 on fibroblast activation and mechanotransduction pathway expression. (I, J) Effect of ATN161 on fibroblast activation in fibronectin‐coated and decellularized matrices. (K) Dosing regimen for TNBS model: Mice received ATN161 (1 mg/kg every two days, i.p.) or equimolar ATN163 from TNBS initiation until endpoint (Control, *n* = 6; TNBS+ATN161, *n* = 6; TNBS+ATN163, *n* = 8). (L) Macroscopic colon appearance in the TNBS model. (M) Masson's trichrome staining in the TNBS model. (N) Quantification of colon length, collagen volume fraction, and submucosal thickness in the TNBS model. (O) Dosing regimen for DSS model (parameters as in K; Control, *n* = 8; DSS+ATN161, *n* = 8; DSS+ATN163, *n* = 8). (P) Macroscopic colon appearance in the DSS model. (Q) Masson's trichrome staining in the DSS model. (R) Quantification of colon length, collagen volume fraction, and submucosal thickness in the DSS model. Human intestinal fibroblasts CCD18Co were used for cell models. Data are presented as mean ±SD. Continuous data were analyzed using Student's t‐test or ANOVA for unpaired groups. Nonparametric distributions were analyzed by Mann‐Whitney/Wilcoxon rank‐sum tests or Kruskal‐Wallis tests, with Dunn's post hoc testing for multiple comparisons. ^*^ *p* < 0.05; ^**^ *p* < 0.01; ^***^ *p* < 0.001. ECM, extracellular matrix; DSS, dextran sulfate sodium salt; TNBS, 2,4,6‐trinitrobenzenesulfonic acid. Scale bar: 20 µm (C); 100 µm (M, Q).

To test if blocking fibronectin‐cell communication could affect cellular activation status, we used ATN161 (Ac‐PHSCN‐NH_2_), a characterized α5β1 antagonist that can competitively inhibit binding between fibronectin's synergistic PSHRN motif and the integrin without disrupting other integrin‐ECM interactions (Figure 7A,B) [28]. Despite reports suggesting ATN161 spares fibronectin‐mediated cell adhesion but impairs downstream signaling, its mechanistic impact remains undefined [29]. Through systematic assays, we demonstrated that ATN161 exerts minimal effects on fibronectin/collagen fibrillogenesis in HIFs, yet significantly reduces cellular activation (Figure 7C,F). This indicates ATN161 specifically disrupts fibronectin‐integrin signaling cascades independent of ECM structural reorganization.

The FAK/SRC and ROCK/RhoA pathways represent primary mechanotransduction routes for integrin‐mediated signaling, upregulating ECM protein expression and enhancing cellular contractility, respectively [30]. Both pathways showed elevated activity under high fibronectin environments, observed not only in the ECM coating model but also in the decellularized model (Figure 7D,E). Administration of ATN161 subsequently downregulated core effector transcripts of these pathways in HIFs (Figure 7G,H). Subsequent assays on fibronectin‐coated matrices confirmed that ATN161 effectively reversed fibronectin‐induced cellular activation (Figure 7I). This normalization effect was consistently replicated in decellularized models with dense fibronectin remnants (Figure 7J). These findings collectively establish that fibronectin engages integrin α5β1 to transduce mechanical cues primarily via the FAK/SRC and ROCK/RhoA axis, driving cellular activation – an effect potently abrogated by disrupting fibronectin‐integrin binding.

### 2.9 Targeting Fibronectin‐Mediated Fibroblast Activation Alone Attenuates Intestinal Fibrosis

We next evaluated whether selectively disrupting fibronectin‐mediated mechanosignaling without altering fibrillogenesis alleviates intestinal fibrosis in vivo. ATN161 and its scrambled peptide control ATN163 were administered in TNBS and DSS models. Unexpectedly, ATN161 substantially attenuated fibrotic indices in the TNBS model, including colon shortening, collagen fraction, and submucosal thickening (Figure 7K–N). In the DSS model, while failing to reverse inflammation‐associated colon shortening, ATN161 still significantly improved collagen deposition and submucosal pathology, suggesting limited anti‐inflammatory efficacy (Figure 7O–R). Furthermore, transcriptomics analysis revealed a significant reduction in ECM‐associated gene expression following ATN161 treatment, mirroring the downregulation observed with pUR4 (Figure S5A–G). These results collectively confirm that fibronectin‐dependent mechanotransduction via integrins drives fibrogenesis, and targeting fibronectin‐integrin binding effectively mitigates intestinal fibrosis.

### 2.10 Inhibition of Fibronectin Ameliorates Established Intestinal Fibrosis

To investigate whether inhibiting fibronectin fibrillogenesis remains effective against established intestinal fibrosis, we first characterized the fibrotic progression in both the DSS and TNBS models. In the DSS model, the submucosa thickened significantly during the acute inflammatory phase, partially regressed after DSS withdrawal, and progressively thickened again over subsequent modeling cycles. Collagen deposition increased steadily, leading to significant fibrosis by the end of the third week (Figure S9A–C). In the TNBS model, significant fibrosis developed starting from the fifth week.

Subsequently, we initiated treatment with pUR4 and ATN161 in the DSS model at the beginning of the fourth week. pUR4 continued to significantly improve the fibrosis score (Figure S9D), whereas the effect of ATN161 was suboptimal (Figure S9E). When treatment was delayed until the seventh week, pUR4 still reduced the fibrosis score, but its efficacy was no longer statistically significant (Figure S9F). These results suggest that therapeutics targeting early‐stage of fibrillogenesis are more effective than those acting on later‐stage targets, and that early intervention with a sufficient treatment course is crucial.

We then tested pUR4 and ATN161 in the TNBS model, initiating treatment at the fifth week against established fibrosis (Figure S9G,H). Both drugs significantly ameliorated intestinal fibrosis in this model. The discrepant outcomes between the two animal models may be attributed to their distinct fibrotic patterns: DSS‐induced fibrosis is often denser and more localized within the submucosa, whereas TNBS‐induced fibrosis is more diffuse and widespread but comparatively less dense. Inhibiting fibronectin assembly may therefore be less effective at reversing a more consolidated and densely developed fibrotic process.

## 3 Discussion

Conventional anti‐fibrotic strategies have focused predominantly on intracellular pathways to reduce ECM deposition. Here, we identify the self‐perpetuating fibroblast‐ECM feedforward loop as a critical pathogenic driver and propose direct extracellular intervention to disrupt ECM accumulation, breaking this cycle. Our findings validate this paradigm, demonstrating fibronectin as a promising molecular target with translational potential for intestinal fibrosis mitigation.

Defining ECM compositional shifts between healthy and fibrotic intestines is critical for identifying key mediators of pathological ECM accumulation. Lin et al. developed a high‐fidelity intestinal decellularization model and identified MFGE8, LTBP1, and VWF as significantly upregulated proteins in clinical fibrotic specimens [11]. Independent mouse intestine matrisome analysis revealed MFGE8 as a top differentially expressed protein. Surprisingly, despite its elevated expression, MFGE8 localized predominantly in epithelial rather than stromal compartments, while established fibrotic markers (fibronectin and collagens) were undetected as differentially abundant—a discordance likely due to technical limits of conventional proteomic pipelines. To resolve this, we implemented a targeted ECM extraction protocol and performed compositional analysis of core structural proteins, ultimately identifying fibronectin as the most significantly altered ECM constituent in fibrotic gut tissues.

While fibronectin's therapeutic potential in fibrosis was shown in hepatic and cardiac studies [16, 17], this work establishes its consistent anti‐fibrotic efficacy in the structurally complex intestinal microenvironment, despite IBD pathogenesis intricacies. Critically, prior research overlooked fibronectin's ECM properties as mechanistic determinants. Herein, we developed two physiologically relevant models to examine fibronectin‐matrix interactions: (1) in vitro fibronectin‐coated substrates constructed under controlled adsorption conditions, and (2) decellularized matrices from fibronectin‐rich ECM produced by HIFs. These models delineate fibronectin‐mediated ECM‐cellular crosstalk, providing novel methodological frameworks for matrix‐targeted fibrosis research.

The DSS model is characterized by a predominant innate immune response and primarily induces damage to the mucosal layer, closely resembling ulcerative colitis in clinical settings [19]. In contrast, the TNBS model features a dominant adaptive immune response and causes transmural damage that penetrates the bowel wall, making it more analogous to clinical Crohn's disease [19]. Together, these two models represent the majority of conditions leading to intestinal fibrosis in clinical practice. The significant alleviation of intestinal fibrosis observed in preventive studies using both models upon fibronectin inhibition underscores the broad applicability and therapeutic potential of targeting fibronectin to mitigate fibrosis.

This consistent anti‐fibrotic effect across two major preclinical models provides a robust foundation for our mechanistic investigation. Our study delineates fibronectin's dual role in mediating intestinal fibrogenesis through ECM fibrillogenesis and mechanotransduction to fibroblasts. We identified targetable molecular domains and selected precision inhibitors: pUR4 demonstrated superior anti‐fibrotic efficacy by suppressing fibronectin fibrillogenesis and collagen disassembly in vivo. Conversely, while R1R2 reduced collagen deposition, it failed to inhibit fibronectin fibrillation and showed no therapeutic benefit. ATN161 effectively blocked integrin‐mediated mechanosignaling without direct ECM effects, yet retained in vivo efficacy. These findings reveal multiple druggable targets on fibronectin with translational promise. Furthermore, PEGylation of pUR4 improved pharmacokinetics without reducing fibronectin affinity [31], indicating feasible optimization for clinical use.

It is noteworthy that although we primarily consider integrin α5β1 as a fibronectin receptor here, its role in fibrosis extends far beyond that. In pancreatic stellate cells, integrin α5β1 interacts with connective tissue growth factor, increasing TGFβ expression and promoting fibrogenesis [32, 33, 34]. Moreover, in hepatic stellate cells, integrin α5β1 binds to cathepsin S, enhancing cell activation and contributing to liver fibrosis [35]. In pulmonary fibrosis, integrin α5β1 interacts with CCL20 to promote lung fibrosis via TGF‐β/Smad activation [36]. While the specific role of integrin α5β1 in intestinal fibrosis has not been well‐documented, studies have indicated that its inhibitor ATN161 can suppress intestinal inflammation by inhibiting angiogenesis [37]. This observation provides valuable complementary context for our finding that ATN161 alleviates intestinal fibrosis. This suggests a potential mechanism whereby integrin α5β1 may exacerbate intestinal inflammation and promote intestinal fibrosis through the promotion of angiogenesis.

Beyond ECM‐fibroblast interactions, fibronectin exerts multifaceted immunomodulatory effects during intestinal fibrosis. Polarized macrophages (M2) and regulatory T cells (Treg) critically drive fibrogenesis via cytokine‐mediated fibroblast activation [38]. Notably, M2 macrophages actively secrete fibronectin, enhancing cellular adhesion while fibronectin‐integrin signaling reciprocally modulates macrophage cytokine profiles and phagocytosis [39, 40, 41]. Treg‐mediated immunomodulation further influences fibrosis progression both directly and through macrophage crosstalk. Correspondingly, fibrillar fibronectin scaffolds also regulate T‐cell trafficking to injury sites [42, 43]. Nevertheless, the spatiotemporal dynamics of fibronectin within gut‐specific immune niches, particularly its interplay with immune subsets in colitis and fibrosis, remain underexplored.

Fibronectin demonstrates context‐dependent roles in fibrosis. At physiologically relevant concentrations, fibronectin may exert protective effects against fibrotic progression. Complete hepatic fibronectin ablation aggravates fibrosis via compensatory mechanisms: TGFβ‐activated stellate cells deposit collagen V scaffolds that facilitate type I/III collagen fibrillogenesis. Concomitantly, fibronectin deficiency increases local TGFβ bioavailability and enhances cellular TGFβ responsiveness [44, 45]. Notably, however, selective disruption of fibronectin assembly using pUR4 or non‐polymerizable fibronectin mutants significantly attenuates liver fibrosis [16]. In our fibroblast‐specific Fn1 knockout model, sustained soluble fibronectin secretion from hepatocytes and intestinal epithelial cells may correlate with intestinal fibroprotection. Furthermore, controlled fibronectin exposure promotes tissue repair, evidenced by the therapeutic efficacy of engineered fibronectin‐small unilamellar vesicles composites in intestinal fibrosis models [9, 46]. These findings underscore the functional heterogeneity of fibronectin in intestinal fibrosis, necessitating further mechanistic investigation.

Beyond fibronectin, numerous targets directly modulate ECM dynamics. Matrix metalloproteinases (MMPs) and tissue inhibitors of metalloproteinases (TIMPs) are complementary protein families whose counterbalancing maintains ECM homeostasis [47, 48]. MMP‐targeted therapies have shown antifibrotic efficacy in cardiac, hepatic, renal, and pulmonary fibrosis models [49, 50, 51]. For intestinal fibrosis, MMP9‐neutralizing antibodies demonstrate therapeutic potential, though precise spatiotemporal control and target specificity dictate efficacy outcomes [52, 53]. Thus, targeted delivery of MMP inhibitors remains a valuable direction, though efficiency requires optimization. Recent studies have also highlighted the A Disintegrin and Metalloproteinase with Thrombospondin motifs (ADAMTS) family as potential regulators of ECM accumulation. Specific ADAMTS members have been shown to attenuate fibrosis in cardiac and renal tissues, as well as in Duchenne muscular dystrophy, likely through ECM degradation and activation of latent TGF‐β [54, 55, 56, 57]. These findings position ADAMTSs as emerging therapeutic targets.

There are several limitations in this study. First, while we identified fibronectin's regulation of ECM deposition and impact on cellular activation, intracellular fibronectin‐mediated signaling remains insufficiently explored. Elucidating how fibronectin modulates intracellular signaling to alter gene expression is critical for understanding its mechanistic role in intestinal fibrosis. Second, although fibronectin inhibitors directly impede ECM accumulation, their direct effects on pathologically activated fibroblasts and inflammatory processes are limited. Combining fibronectin inhibitors with direct fibroblast‐targeting agents and anti‐inflammatory drugs may yield better outcomes. Finally, the absence of clinical trials and the suboptimal route of chronic intraperitoneal administration warrant attention. Developing stable formulations, optimizing delivery methods, and clinically validating fibronectin inhibitors constitute essential next steps.

In conclusion, we propose a novel therapeutic paradigm for intestinal fibrosis by establishing fibronectin as a promising anti‐fibrotic target. Domain‐targeted intervention against fibronectin's functional domains demonstrated robust efficacy in mitigating intestinal fibrosis.

## 4 Experimental Section

### 4.1 Intestinal Tissue Acquisition

Surgical specimens were obtained from patients definitively diagnosed with Crohn's disease via clinical history, presentation, endoscopy, and histopathology, who underwent surgery due to intestinal stricture/obstruction. Patients with strictures attributable to adhesions, Behçet's disease, lymphoma, or unidentified causes were excluded. Paired specimens—fibrotic stiffened intestinal tissue and adjacent macroscopically normal tissue from the surgical resection margin—were fixed in 10% neutral buffered formalin, paraffin‐embedded, and sectioned for subsequent staining. This study was approved by the Qilu Hospital Ethics Committee of Shandong University (KYLL‐2021(KS)‐454).

### 4.2 Intestinal Tissue Decellularization

This protocol was developed from established decellularization methods [11, 58, 59]. Briefly, harvested intestinal tissues were incised along the mesenteric border, and luminal contents were removed. Tissues were bisected longitudinally. One segment was immersed in PBS at room temperature (RT) for preservation. The other segment was agitated in 10% (w/v) sodium dodecyl sulfate solution on an orbital shaker at 100 rpm (RT) for 4–6 h until tissues transitioned from pale‐red to whitish‐translucent. Decellularized segments underwent repeated washing with PBS, followed by incubation in 10% (v/v) Triton X‐100 at 100 rpm (RT) for 1 h. After PBS rinsing, tissues were treated with 100 U/mL DNase I solution under agitation (100 rpm, 37°C) for 30 min. Finally, tissues were rinsed with PBS and stored for subsequent experiments.

### 4.3 Fibronectin Inhibitors

pUR4 [60]: KDQSPLAGESGETEYITEVYGNQQNPVDIDKKLPNETGFSGNMVETEDT; scrambled pUR4: EKGYSKPPVGNEGGDQVDEYDTMSQTKLEDEGNTLISPITFENATEQVN; R1R2 [60]; GLNGENQKEPEQGERGEAGPPLSGLSGNNQGRPSLPGLNGENQKEPEQGERGEAGPP; ATN161 [37]: Ac‐PHSCN‐NH_2_; ATN163 (scrambled ATN161): Ac‐HSPNC‐NH_2_. All peptides were commercially sourced from QYAOBIO (ChinaPeptide Co., Ltd).

### 4.4 Experimental Animals

C57BL/6 mice (wild‐type, 8 weeks old) were supplied by Vital River Laboratory Animal Technology Co., Ltd. (Beijing) and maintained under specific pathogen‐free (SPF) conditions at Shandong University's Model Animal Research Center. Housing parameters included: temperature 18°C–22°C, humidity 50%–60%, 12‐h light/dark cycles, and ad libitum access to food/water. All mice underwent a 7‐day acclimation period prior to experiments. The study protocol was approved by the Ethics Committee of Qilu Hospital, Shandong University (DWLL‐2023‐121). The sample size (*n* ≥ 6) was chosen to match comparable studies in this field [16, 17], which detected significant effects (effect size > 1.0) with similar designs.

### 4.5 Construction of Fn1 Knockout Mice

Col1a2‐CreERT2 transgenic mice (JAX stock #029567) were obtained from Jackson Laboratory. FN1fl/fl mice were generated by Shanghai Model Organisms Centre (Catalog #NM‐CKO‐205170). Genotyping details for both lines are described previously [21, 61, 62]. Genomic DNA from tail biopsies was digested overnight with proteinase K at 55°C. Homogenates were centrifuged at 9,600g for 2 min to pellet debris, followed by DNA precipitation with isopropanol and washing with 70% ethanol. Primers for identifying FN1fl/fl vs. wild‐type alleles are in Table S6.

Conditional knockout mice (Col1a2‐CreERT2; FN1fl/fl) were established by crossing Col1a2‐CreERT2 carriers with FN1fl/fl mice, followed by backcrossing. Mice were housed in SPF facilities (22°C–24°C, 12‐h light/dark cycle) with free access to food/water. Age‐/sex‐matched cohorts were used unless specified.

Before fibrosis induction, 8‐week‐old Fn1fl/fl and Col1a2‐CreERT2 Fn1fl/fl littermates received daily i.p. tamoxifen injections (100 mg/kg) for 4 days. Mice rested for 1 week before subsequent experiments. See Figure S2 for genotyping.

### 4.6 DSS‐Induced Intestinal Fibrosis Model

Modeling mice were randomized and divided into control and DSS groups (including DSS + treatment group). The DSS group received 2% DSS in distilled water ad libitum for 1 week, followed by plain distilled water for 2 weeks, with this cycle repeated three times [19]. Controls received distilled water throughout. Mice were sacrificed at week 9 for intestinal tissue collection. Mice exhibiting periodic weight loss, bloody stools, and diarrhea were deemed to have successful model induction; all such surviving individuals were analyzed.

### 4.7 TNBS‐Induced Intestinal Fibrosis Model

Modeling mice were randomized and divided into control and TNBS groups (including TNBS + treatment group). The TNBS group received weekly intrarectal TNBS: 0.75% in week 1, 1.5% in week 2, and 2.5% from weeks 4 to 7 [19]. Controls received PBS. Sacrifice and tissue collection occurred at week 6. Mice exhibiting periodic weight loss, bloody stools, and diarrhea were deemed to have successful model induction; all such surviving individuals were analyzed.

### 4.8 Fibronectin Inhibitor Treatments

#### 4.8.1 pUR4

From modeling day 1, the DSS+pUR4 group received 25 mg/kg/day pUR4 intraperitoneally (i.p.), the DSS+scrambled pUR4 group received scrambled pUR4 (i.p.), and controls received PBS (i.p.). Treatments continued throughout the experimental period [17].

#### 4.8.2 R1R2

From modeling day 1, the DSS+R1R2 group received 25 mg/kg/day R1R2 (i.p.), the DSS group received PBS (i.p.), and the controls received PBS (i.p.). Administration persisted for the entire modeling duration [60].

#### 4.8.3 ATN161

From modeling day 1, the DSS+ATN161 group received 1 mg/kg ATN161 i.p. every other day, the DSS+ATN163 group received ATN163 (i.p.), and controls received PBS (i.p.). Interventions were maintained until completion of the modeling protocol [37].

### 4.9 Cell Lines and Culture

Human colonic fibroblast CCD18Co (ATCC Cat# CRL‐1459, RRID:CVCL_2379, Lot Number: 70045368, contamination‐free confirmed by ATCC), derived from normal colon tissue of a 2.5‐month‐old female, was cultured in fibroblast medium containing 1% penicillin‐streptomycin, 2% fetal bovine serum, and 1% fibroblast growth factor [63]. Cells were maintained at 37°C with 95% air and 5% CO_2_, passaged at a 1:2 ratio every 3 days using 3–5‐min digestion.

For experiments, cells were seeded at 2 × 10^5^/cm^3^ in culture plates or pre‐coated extracellular matrix (ECM)‐mimetic models. Treatments included 1 ng/ml TGFβ, 50 µg/mL pUR4, 250 µg/mL R1R2, or 10 µg/mL ATN161 as experiment‐specific. To ensure ECM maturation, all cells were harvested after 72 h for RNA/protein extraction or immunofluorescence.

### 4.10 ECM Coating Model

Fibronectin and collagen solutions were prepared at specified concentrations to form a coating mixture. Three hundred microliters of the mixture was added to each well of a 6‐well plate and incubated for 2 h at room temperature to allow ECM component adsorption [64]. Residual liquid was aspirated, and wells were gently rinsed with PBS before use. For coverslips, lysine‐coated coverslips served as the control group, while fibronectin‐coated coverslips were used in the experimental group to ensure consistent cell adhesion.

### 4.11 Cell Decellularization Model

The HIFs were seeded in 12‐well plates at densities of 2.5 × 10^4 cells/well with or without 1 ng/mL TGFβ treatment. Cells were maintained in Fibroblast Medium (2% fetal bovine serum, 1% Fibroblast Growth Supplement, 1% penicillin/streptomycin) at 37°C with 5% CO_2_ for 3 days. For the thick‐layer decellularization model, HIFs were seeded in 12‐well plates at densities of 1 × 10^5 cells/well with or without 1 ng/mL TGFβ, and treated for 5 days. Plates were washed with PBS and treated with 0.05 mm EGTA on ice with agitation (90 rpm) for 30 min. After PBS rinsing, samples were exposed to 0.01% (w/v) sodium deoxycholate on ice with agitation (90 rpm) for 15 min. Following additional PBS rinses, tissues were treated with 50 U/mL DNase I under agitation (60 rpm, 37°C) for 30 min, rinsed with PBS, and reserved for subsequent analysis [65, 66].

### 4.12 Statistical Analysis

Continuous data were analyzed using Student's t‐test or ANOVA for unpaired groups. Nonparametric distributions were analyzed by Mann‐Whitney/Wilcoxon rank‐sum tests or Kruskal‐Wallis tests, with Dunn's post hoc testing for multiple comparisons. Results are reported as mean ± SD, with statistical significance defined as p < 0.05. All analyses were conducted in GraphPad Prism8 (GraphPad Software, San Diego, California, USA).

## Author Contributions

W.M., MD (Conceptualization: Lead; Formal analysis: Lead; Investigation: Lead; Methodology: Lead; Visualization: Lead; Writing – original draft: Lead; Writing – review & editing: Lead). S.Y., MS (Formal analysis: Supporting; Investigation: Equal; Writing – review & editing: Supporting). T.W., PhD (Investigation: Equal; Writing – review & editing: Supporting, Resources: Supporting). D.Z., PhD (Investigation: Supporting; Methodology: Supporting). H.W., MD (Investigation: Supporting; Methodology: Supporting). S.F., PhD (Investigation: Supporting; Methodology: Supporting). X.W., MD (Investigation: Supporting; Methodology: Supporting). L.L., PhD (Project administration: Supporting; Resources: Supporting; Supervision: Supporting). X.Z., PhD (Funding acquisition: Equal; Supervision: Supporting). Y.L., PhD (Funding acquisition: Equal; Supervision: Supporting). J.L., PhD (Conceptualization: Lead; Funding acquisition: Lead; Resources: Lead; Methodology: Equal; Supervision: Lead; Writing – review & editing: Lead).

## Funding

This study was funded by the National Natural Science Foundation of China (82370524 and 82100535) and the Taishan Scholar Project of Shandong Province (tsqn202408340).

## Conflicts of Interest

The authors declare no conflicts of interest.

## Ethics

This study was approved by the Qilu Hospital Ethics Committee of Shandong University (KYLL‐2021(KS)‐454), and written informed consent for sample collection was obtained from all patients. The animal experiment was approved by the Ethics Committee on Animal Experiment of Shandong University Qilu Hospital (DWLL‐2023‐121).

## Supporting information




**Supporting File**: advs74166‐sup‐0001‐SuppMat.docx.

## Data Availability

The proteomics data of clinical specimens originated form published article. They could be found in the ProteomeXchange Consortium (https://proteomecentral.proteomexchange.org) via the PRIDE partner repository, with the dataset identifier PXD012284. The mass spectrometry proteomics data have been deposited to the Proteome Xchange Consortium (https://proteomecentral.proteomexchange.org) via the iProX partner repository with the dataset identifier PXD066838. The raw sequence data of RNA‐seq reported in this paper have been deposited in the Genome Sequence Archive (Genomics, Proteomics & Bioinformatics 2021) in the National Genomics Data Center (Nucleic Acids Res 2022), China National Center for Bioinformation / Beijing Institute of Genomics, Chinese Academy of Sciences (GSA: CRA028569) that are publicly accessible at https://ngdc.cncb.ac.cn/gsa.

## References

[1] S. D'Alessio , F. Ungaro , D. Noviello , S. Lovisa , L. Peyrin‐Biroulet , and S. Danese , “Revisiting Fibrosis in Inflammatory Bowel Disease: The Gut Thickens,” Nature Reviews Gastroenterology & Hepatology 19, no. 3 (2022): 169–184. 34876680 10.1038/s41575-021-00543-0

[2] S.‐N. Lin , R. Mao , C. Qian , et al., “Development of Antifibrotic Therapy for Stricturing Crohn's Disease: Lessons from Randomized Trials in Other Fibrotic Diseases,” Physiological Reviews 102, no. 2 (2022): 605–652. 34569264 10.1152/physrev.00005.2021 PMC8742742

[3] F. Rieder , P. K. Mukherjee , W. J. Massey , Y. Wang , and C. Fiocchi , “Fibrosis in IBD: From Pathogenesis to Therapeutic Targets,” Gut 73, no. 5 (2024): 854–866. 38233198 10.1136/gutjnl-2023-329963 PMC10997492

[4] L. A. Johnson , E. S. Rodansky , K. L. Sauder , et al., “Matrix Stiffness Corresponding to Strictured Bowel Induces a Fibrogenic Response in Human Colonic Fibroblasts,” Inflammatory Bowel Diseases 19, no. 5 (2013): 891–903. 23502354 10.1097/MIB.0b013e3182813297 PMC3766259

[5] A. R. Rackow , D. J. Nagel , C. McCarthy , et al., “The Self‐fulfilling Prophecy of Pulmonary Fibrosis: A Selective Inspection of Pathological Signalling Loops,” European Respiratory Journal 56, no. 5 (2020): 2000075. 32943406 10.1183/13993003.00075-2020 PMC7931159

[6] H. Liu , Y. Hong , H. Chen , et al., “Dual Activation of GCGR/GLP1R Signaling Ameliorates Intestinal Fibrosis via Metabolic Regulation of Histone H3K9 Lactylation in Epithelial Cells,” Acta Pharmaceutica Sinica B 15, no. 1 (2025): 278–295. 40041889 10.1016/j.apsb.2024.11.017 PMC11873616

[7] Y. Zhang , J. Wang , H. Sun , et al., “TWIST1+FAP+ Fibroblasts in the Pathogenesis of Intestinal Fibrosis in Crohn's Disease,” Journal of Clinical Investigation 134, no. 18 (2024): 179472. 10.1172/JCI179472 PMC11405050 39024569

[8] J. D. Bonadio , G. Bashiri , P. Halligan , M. Kegel , F. Ahmed , and K. Wang , “Delivery Technologies for Therapeutic Targeting of Fibronectin in Autoimmunity and Fibrosis Applications,” Advanced Drug Delivery Reviews 209 (2024): 115303. 38588958 10.1016/j.addr.2024.115303 PMC11111362

[9] J. Patten and K. Wang , “Fibronectin in Development and Wound Healing,” Advanced Drug Delivery Reviews 170 (2021): 353–368. 32961203 10.1016/j.addr.2020.09.005

[10] Y. Chen , J. Li , X. Zhang , et al., “Mesenteric Adipose‐Derived Exosomal TINAGL1 Enhances Intestinal Fibrosis in Crohn's Disease via SMAD4,” Journal of Advanced Research 70 (2025): 139–158. 38750695 10.1016/j.jare.2024.05.016 PMC11976418

[11] S. Lin , J. Wang , P. K. Mukherjee , et al., “Milk Fat Globule‐epidermal Growth Factor 8 (MFGE8) Prevents Intestinal Fibrosis,” Gut 73, no. 7 (2024): 1110–1123. 38378253 10.1136/gutjnl-2022-328608 PMC11248270

[12] K. E. Kubow , R. Vukmirovic , L. Zhe , et al., “Mechanical Forces Regulate the Interactions of Fibronectin and Collagen I in Extracellular Matrix,” Nature Communications 6 (2015): 8026. 10.1038/ncomms9026 PMC4539566 26272817

[13] J. Sottile , F. Shi , I. Rublyevska , H.‐Y. Chiang , J. Lust , and J. Chandler , “Fibronectin‐dependent Collagen I Deposition Modulates the Cell Response to Fibronectin,” American Journal of Physiology‐Cell Physiology 293, no. 6 (2007): C1934–C1946. 17928541 10.1152/ajpcell.00130.2007

[14] N. S. Greaves , K. J. Ashcroft , M. Baguneid , and A. Bayat , “Current Understanding of Molecular and Cellular Mechanisms in Fibroplasia and Angiogenesis during Acute Wound Healing,” Journal of Dermatological Science 72, no. 3 (2013): 206–217. 23958517 10.1016/j.jdermsci.2013.07.008

[15] J. A. McDonald , D. G. Kelley , and T. J. Broekelmann , “Role of Fibronectin in Collagen Deposition: Fab' to the Gelatin‐binding Domain of Fibronectin Inhibits both Fibronectin and Collagen Organization in Fibroblast Extracellular Matrix,” The Journal of Cell Biology 92, no. 2 (1982): 485–492. 7061591 10.1083/jcb.92.2.485 PMC2112086

[16] E. Altrock , C. Sens , C. Wuerfel , et al., “Inhibition of Fibronectin Deposition Improves Experimental Liver Fibrosis,” Journal of Hepatology 62, no. 3 (2015): 625–633. 24946284 10.1016/j.jhep.2014.06.010

[17] I. Valiente‐Alandi , S. J. Potter , A. M. Salvador , et al., “Inhibiting Fibronectin Attenuates Fibrosis and Improves Cardiac Function in a Model of Heart Failure,” Circulation 138, no. 12 (2018): 1236–1252. 29653926 10.1161/CIRCULATIONAHA.118.034609 PMC6186194

[18] N. Pierre , C. Salée , C. Massot , et al., “Proteomics Highlights Common and Distinct Pathophysiological Processes Associated with Ileal and Colonic Ulcers in Crohn's Disease,” Journal of Crohn's and Colitis 14, no. 2 (2020): 205–215. 10.1093/ecco-jcc/jjz130 31282946

[19] S. Wirtz , V. Popp , M. Kindermann , et al., “Chemically Induced Mouse Models of Acute and Chronic Intestinal Inflammation,” Nature Protocols 12, no. 7 (2017): 1295–1309. 28569761 10.1038/nprot.2017.044

[20] J. Keski‐Oja and G. J. Todaro , “Specific Effects of Fibronectin‐Releasing Peptides on the Extracellular Matrices of Cultured Human Fibroblasts,” Cancer Research 40, no. 12 (1980): 4722–4727. 7002296

[21] X. Yuan , S. Yang , W. Li , et al., “Construction of Fibronectin Conditional Gene Knock‐out Mice and the Effect of Fibronectin Gene Knockout on Hematopoietic, Biochemical and Immune Parameters in Mice,” PeerJ 8 (2020): 10224. 10.7717/peerj.10224 PMC7605225 33194415

[22] C. Arriagada , E. Lin , M. Schonning , and S. Astrof , “Mesodermal Fibronectin Controls Cell Shape, Polarity, and Mechanotransduction in the Second Heart Field during Cardiac Outflow Tract Development,” Developmental Cell 60, no. 1 (2025): 62–84. 39413783 10.1016/j.devcel.2024.09.017 PMC11706711

[23] B. R. Tomasini‐Johansson , N. R. Kaufman , M. G. Ensenberger , V. Ozeri , E. Hanski , and D. F. Mosher , “A 49‐residue Peptide from Adhesin F1 of Streptococcus pyogenes Inhibits Fibronectin Matrix Assembly,” Journal of Biological Chemistry 276, no. 26 (2001): 23430–23439. 11323441 10.1074/jbc.M103467200

[24] H. Lindmark and B. Guss , “SFS, a Novel Fibronectin‐Binding Protein from Streptococcus equi, Inhibits the Binding between Fibronectin and Collagen,” Infection and Immunity 67, no. 5 (1999): 2383–2388. 10225899 10.1128/iai.67.5.2383-2388.1999 PMC115982

[25] E. S. White , D. L. Livant , S. Markwart , and D. A. Arenberg , “Monocyte‐Fibronectin Interactions, Via α5β1 Integrin, Induce Expression of CXC Chemokine‐Dependent Angiogenic Activity,” The Journal of Immunology 167, no. 9 (2001): 5362–5366. 11673553 10.4049/jimmunol.167.9.5362

[26] R. J. Slack , S. J. F. Macdonald , J. A. Roper , R. G. Jenkins , and R. J. D. Hatley , “Emerging Therapeutic Opportunities for Integrin Inhibitors,” Nature Reviews Drug Discovery 21, no. 1 (2022): 60–78. 34535788 10.1038/s41573-021-00284-4 PMC8446727

[27] P. Singh , C. Carraher , and J. E. Schwarzbauer , “Assembly of Fibronectin Extracellular Matrix,” Annual Review of Cell and Developmental Biology 26 (2010): 397–419. 10.1146/annurev-cellbio-100109-104020 PMC3628685 20690820

[28] D. L. Livant , et al., “Anti‐Invasive, Antitumorigenic, and Antimetastatic Activities of the PHSCN Sequence in Prostate Carcinoma,” Cancer Research 60, no. 2 (2000): 309–320. 10667582

[29] O. Stoeltzing , W. Liu , N. Reinmuth , et al., “Inhibition of Integrin α 5 β 1 Function With A Small Peptide (ATN‐161) Plus Continuous 5‐FU Infusion Reduces Colorectal Liver Metastases And Improves Survival In Mice,” International Journal of Cancer 104, no. 4 (2003): 496–503. 12584749 10.1002/ijc.10958

[30] J. Cooper and F. G. Giancotti , “Integrin Signaling in Cancer: Mechanotransduction, Stemness, Epithelial Plasticity, and Therapeutic Resistance,” Cancer Cell 35, no. 3 (2019): 347–367. 30889378 10.1016/j.ccell.2019.01.007 PMC6684107

[31] P. Zbyszynski , B. R. Tomasini‐Johansson , D. M. Peters , and G. S. Kwon , “Characterization of the PEGylated Functional Upstream Domain Peptide (PEG‐FUD): A Potent Fibronectin Assembly Inhibitor with Potential as an Anti‐Fibrotic Therapeutic,” Pharmaceutical Research 35, no. 7 (2018): 126. 29691664 10.1007/s11095-018-2412-7 PMC6186450

[32] R. Gao and D. R. Brigstock , “Connective Tissue Growth Factor (CCN2) in Rat Pancreatic Stellate Cell Function: Integrin α5β1 as a Novel CCN2 Receptor,” Gastroenterology 129, no. 3 (2005): 1019–1030. 16143139 10.1053/j.gastro.2005.06.067

[33] Y.‐C. Chen , T.‐Y. Chuang , C.‐W. Liu , et al., “Particulate Matters Increase Epithelial‐mesenchymal Transition and Lung Fibrosis through the ETS‐1/NF‐κB‐dependent Pathway in Lung Epithelial Cells,” Particle and Fibre Toxicology 17, no. 1 (2020): 41. 32799885 10.1186/s12989-020-00373-z PMC7429884

[34] R. Gao and D. R. Brigstock , “A Novel Integrin α 5 β 1 Binding Domain In Module 4 Of Connective Tissue Growth Factor (CCN2/CTGF) Promotes Adhesion And Migration Of Activated Pancreatic Stellate Cells,” Gut 55, no. 6 (2006): 856–862. 16361307 10.1136/gut.2005.079178 PMC1856205

[35] T. Zuo , Q. Xie , J. Liu , et al., “Macrophage‐Derived Cathepsin S Remodels the Extracellular Matrix to Promote Liver Fibrogenesis,” Gastroenterology 165, no. 3 (2023): 746–761. 37263311 10.1053/j.gastro.2023.05.039

[36] S. Liu , Q. Wang , J. Min , et al., “The CCL20–Integrin α5β1 Interaction Enhances TGF‐β/Smad Signaling To Promote Fibroblast Activation In Pulmonary Fibrosis,” Nature Communications 16, no. 1 (2025): 9183. 10.1038/s41467-025-64211-6 PMC12532996 41102188

[37] S. Danese , M. Sans , D. M. Spencer , et al., “Angiogenesis Blockade as a New Therapeutic Approach to Experimental Colitis,” Gut 56, no. 6 (2007): 855–862. 17170016 10.1136/gut.2006.114314 PMC1954843

[38] S. D. Blystone , L. K. Weston , and J. E. Kaplan , “Fibronectin Dependent Macrophage Fibrin Binding,” Blood 78 (1991): 2900–2907. 1954378

[39] C. Logie , T. van Schaik , T. Pompe , and K. Pietsch , “Fibronectin‐Functionalization of 3D Collagen Networks Supports Immune Tolerance and Inflammation Suppression in Human Monocyte‐derived Macrophages,” Biomaterials 268 (2021): 120498. 33276199 10.1016/j.biomaterials.2020.120498

[40] A. H. Sikkema , J. M. J. Stoffels , P. Wang , et al., “Fibronectin Aggregates Promote Features of a Classically and Alternatively Activated Phenotype in Macrophages,” Journal of Neuroinflammation 15, no. 1 (2018): 218. 30071854 10.1186/s12974-018-1238-x PMC6091019

[41] K. Alitalo , T. Hovi , and A. Vaheri , “Fibronectin Is Produced by Human Macrophages,” The Journal of Experimental Medicine 151, no. 3 (1980): 602–613. 7359083 10.1084/jem.151.3.602 PMC2185817

[42] Z. Li , D. Li , A. Tsun , and B. Li , “FOXP3^+^ Regulatory T Cells and Their Functional Regulation,” Cellular & Molecular Immunology 12, no. 5 (2015): 558–565. 25683611 10.1038/cmi.2015.10 PMC4579651

[43] N. R. J. Fernandes , N. S. Reilly , D. C. Schrock , D. C. Hocking , P. W. Oakes , and D. J. Fowell , “CD4^+^ T Cell Interstitial Migration Controlled by Fibronectin in the Inflamed Skin,” Frontiers in Immunology 11 (2020): 1501. 32793204 10.3389/fimmu.2020.01501 PMC7393769

[44] K. Moriya , E. Bae , K. Honda , et al., “A Fibronectin‐independent Mechanism of Collagen Fibrillogenesis in Adult Liver Remodeling,” Gastroenterology 140, no. 5 (2011): 1653–1663. 21320502 10.1053/j.gastro.2011.02.005 PMC3081910

[45] N. Kawelke , M. Vasel , C. Sens , A. von Au , S. Dooley , and I. A. Nakchbandi , “Fibronectin Protects from Excessive Liver Fibrosis by Modulating the Availability of and Responsiveness of Stellate Cells to Active TGF‐β,” PLoS ONE 6, no. 11 (2011): 28181. 10.1371/journal.pone.0028181 PMC3225392 22140539

[46] K. Y. Lee , H. T. Nguyen , A. Setiawati , et al., “An Extracellular Matrix‐Liposome Composite, A Novel Extracellular Matrix Delivery System for Accelerated Tissue Regeneration,” Advanced Healthcare Materials 11, no. 4 (2022): 2101599. 10.1002/adhm.202101599 34800312

[47] K. Li , F. R. Tay , and C. K. Y. Yiu , “The Past, Present and Future Perspectives of Matrix Metalloproteinase Inhibitors,” Pharmacology & Therapeutics 207 (2020): 107465. 31863819 10.1016/j.pharmthera.2019.107465

[48] M. Li , et al., “A Versatile Platform Based on Matrix Metalloproteinase‐Sensitive Peptides for Novel Diagnostic and Therapeutic Strategies in Arthritis,” Bioactive Materials 47 (2025): 100–120. 39897588 10.1016/j.bioactmat.2025.01.011 PMC11787566

[49] S. Mahalanobish , S. Saha , S. Dutta , and P. C. Sil , “Matrix Metalloproteinase: An Upcoming Therapeutic Approach for Idiopathic Pulmonary Fibrosis,” Pharmacological Research 152 (2020): 104591. 31837390 10.1016/j.phrs.2019.104591

[50] L. Shan , F. Wang , D. Zhai , X. Meng , J. Liu , and X. Lv , “Matrix Metalloproteinases Induce Extracellular Matrix Degradation through Various Pathways to Alleviate Hepatic Fibrosis,” Biomedicine & Pharmacotherapy 161 (2023): 114472. 37002573 10.1016/j.biopha.2023.114472

[51] H. Toba and M. L. Lindsey , “Extracellular Matrix Roles in Cardiorenal Fibrosis: Potential Therapeutic Targets for CVD and CKD in the Elderly,” Pharmacology & Therapeutics 193 (2019): 99–120. 30149103 10.1016/j.pharmthera.2018.08.014 PMC6309764

[52] E. A. Baltazar‐García , et al., “Deflamin Attenuated Lung Tissue Damage in an Ozone‐Induced COPD Murine Model by Regulating MMP‐9 Catalytic Activity,” International Journal of Molecular Sciences 25, no. 10 (2024): 5063. 38791100 10.3390/ijms25105063 PMC11121448

[53] L. Goffin , S. Fagagnini , A. Vicari , et al., “Anti‐MMP‐9 Antibody,” Inflammatory Bowel Diseases 22, no. 9 (2016): 2041–2057. 27542125 10.1097/MIB.0000000000000863

[54] J. Dulos , D. A. Debruin , E. van der Aar , et al., “ADAMTS‐5 Inhibition Reduces Muscle Inflammation and Fibrosis and Improves Function in Mouse Models of Duchenne Muscular Dystrophy,” Science Translational Medicine 17, no. 803 (2025): ado2112. 10.1126/scitranslmed.ado2112 40531968

[55] M. M. Khan , G. Galea , J. Jung , et al., “Dextromethorphan Inhibits Collagen and Collagen‐like Cargo Secretion to Ameliorate Lung Fibrosis,” Science Translational Medicine 16, no. 778 (2024): adj3087. 10.1126/scitranslmed.adj3087 39693409

[56] F. Link , Y. Li , J. Zhao , et al., “ECM1 attenuates Hepatic Fibrosis by Interfering with Mediators of Latent TGF‐β1 Activation,” Gut 74, no. 3 (2025): 424–439. 39448254 10.1136/gutjnl-2024-333213

[57] M. Vistnes , P. M. Erusappan , A. Sasi , et al., “Inhibition of the Extracellular Enzyme A Disintegrin and Metalloprotease with Thrombospondin Motif 4 Prevents Cardiac Fibrosis and Dysfunction,” Cardiovascular Research 119, no. 10 (2023): 1915–1927. 37216909 10.1093/cvr/cvad078 PMC10439713

[58] A.‐M. Diedrich , A. Daneshgar , P. Tang , et al., “Proteomic Analysis of Decellularized Mice Liver and Kidney Extracellular Matrices,” Journal of Biological Engineering 18, no. 1 (2024): 17. 38389090 10.1186/s13036-024-00413-8 PMC10885605

[59] P. Maghsoudlou , G. Totonelli , S. P. Loukogeorgakis , S. Eaton , and P. De Coppi , “A Decellularization Methodology for the Production of a Natural Acellular Intestinal Matrix,” Journal of Visualized Experiments, no. 80 (2013): 50658. 24145913 10.3791/50658 PMC3923547

[60] H. Ghura , M. Keimer , A. von Au , N. Hackl , V. Klemis , and I. A. Nakchbandi , “Inhibition of Fibronectin Accumulation Suppresses Tumor Growth,” Neoplasia 23, no. 9 (2021): 837–850. 34298233 10.1016/j.neo.2021.06.012 PMC8322122

[61] N. Wu , H. Sun , X. Zhao , et al., “MAP3K2‐regulated Intestinal Stromal Cells Define a Distinct Stem Cell Niche,” Nature 592, no. 7855 (2021): 606–610. 33658717 10.1038/s41586-021-03283-y

[62] X. Tian , L. He , K. Liu , et al., “Generation of a Self‐Cleaved Inducible Cre Recombinase For Efficient Temporal Genetic Manipulation,” The EMBO Journal 39, no. 4 (2020): EMBJ2019102675. 10.15252/embj.2019102675 PMC7024834 31943281

[63] H. Xiao , et al., “Adiponectin Deficiency Prevents Chronic Colitis‐Associated Colonic Fibrosis via Inhibiting CXCL13 Production,” Journal of Advanced Research 76 (2024): 639–653. 39725008 10.1016/j.jare.2024.12.032 PMC12793756

[64] P. Lu , Z. Chen , M. Wu , et al., “Type I Collagen Extracellular Matrix Facilitates Nerve Regeneration via the Construction of a Favourable Microenvironment,” Burns & Trauma 12 (2024): tkae049. 39659559 10.1093/burnst/tkae049 PMC11631217

[65] Y. Peng , L. Li , J. Shang , et al., “Macrophage Promotes Fibroblast Activation and Kidney Fibrosis by Assembling a Vitronectin‐Enriched Microenvironment,” Theranostics 13, no. 11 (2023): 3897–3913. 37441594 10.7150/thno.85250 PMC10334827

[66] Q. Xing , K. Yates , M. Tahtinen , E. Shearier , Z. Qian , and F. Zhao , “Decellularization of Fibroblast Cell Sheets for Natural Extracellular Matrix Scaffold Preparation,” Tissue Engineering Part C: Methods 21, no. 1 (2015): 77–87. 24866751 10.1089/ten.tec.2013.0666 PMC4291209

