# Supplementary material for: Direct Extracellular Matrix Modulation Attenuates Intestinal Fibrosis via a Fibronectin‐Targeted Approach
Source: Adv Sci (Weinh). 2026 Jan 30;13(20):e19433. doi: 10.1002/advs.202519433 (PMC13067841; doi:10.1002/advs.202519433)
Supplement: Supplementary file 1 — Supporting File: advs74166‐sup‐0001‐SuppMat.docx. [file ADVS-13-e19433-s001.docx]

**Supporting information**

**Title: Direct extracellular matrix modulation attenuates intestinal fibrosis via a fibronectin-targeted approach**

Wenlong Ma1, Siyu Yang1, Tengkai Wang1, Di Zhang12, Hewei Wu1, Shichen Fu1, Xiaohan Wan1, Lixiang Li134, Xiuli Zuo1*, Yanqing Li1*, Jiaoyang Lu15*

1.Department of Gastroenterology, Qilu Hospital of Shandong University, Jinan, Shandong, People’s Republic of China

2.Department of Medical Oncology, Qilu Hospital of Shandong University, Jinan, Shandong, People’s Republic of China

3. Laboratory of Translational Gastroenterology, Qilu Hospital of Shandong University,

Jinan, Shandong, People’s Republic of China

4. Shandong Provincial Clinical Research Center for Digestive Disease, Jinan, Shandong, People’s Republic of China

5.Medical Integration and Practice Center, Shandong University, Jinan, Shandong,

People’s Republic of China

Wenlong Ma, Siyu Yang and Tengkai Wang contributed equally to this work

Corresponding author: Jiaoyang Lu, lujiaoyang@sdu.edu.cn; Yanqing Li, liyanqing@sdu.edu.cn; Xiuli Zuo, zuoxiuli@sdu.edu.cn. Department of Gastroenterology, Qilu Hospital,Shandong University, 107 West Wen Hua Road, Jinan, Shandong 250012, China**.**

**Supplementary results**

**Fibronectin Structure and Assembly**

Fibronectin is an extracellular matrix glycoprotein existing predominantly as homodimers^1^. Each subunit contains conserved modular domains organized into three repeating types (Type I, II, and III repeats). Fibronectin adopts two physiological forms: soluble plasma fibronectin secreted by hepatocytes and insoluble cellular fibronectin deposited in the extracellular matrix (ECM) by fibroblasts and activated myofibroblasts. Insoluble ECM assembly requires core functional domains: the fibronectin assembly domain (Type I repeats 1–5), collagen-binding domain (Type I repeats 6–9 and Type II repeats 1–2), synergy site PHSRN (Type III repeat 9), and RGD motif (Type III repeat 10). During assembly, cell-surface integrins (primarily α5β1; alternatively αvβ1/αvβ3 in pathology) bind the RGD motif to anchor fibronectin, with PHSRN cooperatively enhancing this process. Subsequent exposure of the fibronectin assembly domain initiates trans-dimerization with adjacent fibronectin molecules, forming nascent fibronectin networks. Collagen-binding domains integrate these networks with other ECM components (e.g., collagens), while disulfide bonding stabilizes the mature pericellular matrix architecture.

**Binding Site of pUR4**

pUR4 acts as a fibronectin assembly inhibitor by targeting the fibronectin1 assembly domain. While early studies identified its inhibitory function, the precise mechanism remained unresolved due to technical limitations in fibronectin fragmentation^2^. Our molecular docking reveals that pUR4 embeds into the fibronectin tertiary structure, directly engaging 45 residues spanning amino acids 98–539 (encompassing Type I repeats 2–9 and Type II repeats 1–2). This interaction sterically blocks the entire fibronectin1 assembly domain, with robust validation metrics: iPTM score 0.6 (>0.5 threshold for reliability), binding interface area 3402.4 Å² (>1500 Å² indicating strong interaction), and Binding Free Energy (BFE) -16.6 kcal/mol (<-12 kcal/mol confirming high-affinity binding).

**Binding Site of R1R2**

R1R2 inhibits collagen-fibronectin assembly by targeting Type I repeats 8–9. Previous technical limitations in fibronectin fragmentation obscured its precise binding mechanism^3, 4^. Our molecular docking demonstrates that R1R2 partially embeds into the fibronectin tertiary structure, directly engaging 23 residues within amino acids 439–599 (spanning Type I repeats 7–8 and Type II repeat 2). This interaction sterically blocks the collagen-binding domain, with validation metrics confirming reliability: iPTM score 0.52 (>0.5 threshold), binding interface area 2007.0 Å² (>1500 Å² indicating strong interaction), and BFE -10.0 kcal/mol (range -8 to -12 kcal/mol defining moderate binding affinity).

**Integrin α5β1 Inhibitor Selection**

While RGD-motif inhibitors efficiently block integrin-fibronectin binding, their broad specificity toward multiple extracellular matrix proteins nevertheless obscures mechanistic interpretation^5, 6^. To address this, we instead employed ATN161, a moderate-affinity inhibitor that targets the fibronectin-specific PHSRN synergy site. It is true that ATN161 is frequently referenced as an angiogenesis inhibitor and limited mechanistic studies exist on it^7, 8^; however, sparse literature indicates that it reduces fibronectin-mediated cell adhesion but does not necessarily reduce integrin-dependent adhesion^9, 10^ . Significantly, our data demonstrate that while ATN161 does not impede fibronectin matrix assembly—which is distinct from RGD inhibitors—it nonetheless significantly attenuates cellular activation and mechanosignaling. Moreover, consistent with these attenuated fibrogenic responses, ATN161 ameliorated intestinal fibrosis in vivo. Collectively, these findings indicate that ATN161 selectively disrupts integrin-mediated mechanotransduction without compromising fibronectin matrix polymerization.

**Bulk RNA-seq Analysis of pUR4 and ATN161 in Fibrotic Intestine**

Transcriptomic profiling and PCA revealed no distinct clustering among TNBS-induced fibrotic intestines versus those treated with ATN161 or pUR4. Venn analysis identified 410 differentially expressed genes (DEGs) in pUR4-treated samples and 170 DEGs in ATN161-treated samples relative to TNBS controls, with 57 shared DEGs. In down-regulated GO terms (top 10 enrichment), pUR4 altered genes associated with external side of plasma membrane, extracellular space, extracellular region, plasma membrane, cell surface, extracellular matrix, and side of membrane. ATN161 impacted extracellular region, extracellular space, side of membrane, and cell surface. For down-regulated KEGG pathways (top 10), pUR4 modulated ECM-receptor interaction and Cell adhesion molecules, while ATN161 affected Cell adhesion molecules. These findings indicate both inhibitors suppress pathological ECM overdeposition in intestinal fibrosis, disrupt profibrotic transmembrane signaling, and ameliorate fibrotic progression.

**Supplementary Materials and Methods**

**Histological Section Preparation and Evaluation**

Fresh intestinal tissues from Crohn’s disease (CD) patients and mouse models were fixed in 4% paraformaldehyde for 24 hours, transferred to 70% ethanol for 24 hours, and paraffin-embedded. Formalin-fixed paraffin-embedded CD tissues were sectioned at 4 μm for hematoxylin and eosin (H&E), Sirius red, and Masson’s trichrome staining. Sections were scanned using an Olympus VS120 slide scanner, with three random fields per slide selected for quantitative analysis. The thickness of the mucosal layer (from the epithelial surface to the muscularis mucosae) and the muscularis propria (from the muscularis mucosae to the boundary of the serosa or the outer longitudinal muscle layer) was measured by drawing lines perpendicular to the mucosal surface using NDPviewer2. Two specialized IBD pathologists evaluated pathological scores^11^ and thickness of the mucosa, submucosa, and muscularis propria layers. The criteria for inflammation scoring: inflammatory cell infiltration in the lamina propria (occasional inflammatory cells = 0; increased inflammatory cells = 1; confluence of inflammatory cells extending to the submucosa = 2; transmural extension = 3), tissue damage (no mucosal damage = 0; lymphoepithelial lesions = 1; surface mucosal erosion = 2; extensive mucosal damage and extension into deeper structures of the bowel wall = 3), and the extent of intestinal involvement (focal, 0-30% = 0; multifocal to regional, 30-60% = 1; diffuse, >60% = 2). Sirius red stanning images under polarized light were captured by research-grade cold and hot stage polarizing microscope “Axio Scope.A 1”A Pol. Collagen volume was quantified using ImageJ; the initial macro for each batch was applied uniformly to all subsequent samples within the same batch.

**Immunohistochemistry (IHC) and Immunofluorescence (IF)**

Tissue sections were baked at 65°C for 30 min, deparaffinized in xylene (twice, 10 min each), and rehydrated through graded alcohols: 100% ethanol (twice, 5 min each), 95% ethanol (2 min), and 70% ethanol (2 min), followed by distilled water rinse (1 min). Antigen retrieval was performed in citrate (pH 6.0) or EDTA (pH 8.0) buffer by boiling for 3 min and maintaining at sub-boiling for 20 min. After cooling to room temperature (RT), sections were washed with PBS (thrice, 3 min each). Endogenous peroxidase activity was blocked with blocking buffer (RT, 10 min). Primary antibodies (100 μL, volume-adjusted for tissue area) were incubated at 37°C for 60 min or 4°C overnight. Following PBS washes, sections were incubated with a reaction enhancer (37°C, 20 min), then horseradish peroxidase-conjugated secondary antibody (37°C, 30 min). Color development used freshly diluted DAB (1:50), monitored microscopically. Sections were rinsed in tap water, counterstained with hematoxylin (1 min), differentiated in 1% acid-alcohol (1 sec), blued in running water (10 min), dehydrated, cleared in xylene, and mounted with neutral resin.

For IF, endogenous peroxidase blocking was omitted. Sections were blocked with donkey serum (30 min) before primary antibody incubation. After primary antibody incubation, fluorophore-conjugated secondary antibodies were applied (RT, 60 min), followed by DAPI nuclear counterstaining and mounting.

All sections were scanned using an Olympus VS120 (IHC) or VS200 (IF) slide scanner. Three random fields per section were selected for quantitative analysis. IHC quantification utilized ImageJ’s IHC Toolbox with batch-specific macros standardized using the first sample of each batch. IF colocalization analysis was performed using ImageJ.

**Transmission Electron Microscopy (TEM) Assessment of Intestinal Fibrosis**

Freshly resected intestinal tissues were rapidly rinsed in PBS and immediately fixed in 3% glutaraldehyde (pH 7.4). Tissue blocks were uniformly trimmed to 1 × 1 × 3 mm sections oriented in the same direction^12^. Samples were processed using conventional TEM protocols: sequential rinsing, post-fixation in 1% osmium tetroxide (OsO₄), dehydration, epoxy resin (Epon 812) embedding, and polymerization. Semi-thin and ultra-thin sections were prepared from basal and middle regions, then double-stained with uranyl acetate and lead citrate^13^. Grids were examined under a JEOL-1200EX transmission electron microscope at JiNan WeiYa Bio-Technology Co., Ltd. (Jinan, China).

**Label-Free Proteomics Workflow**

Protein Extraction: Samples were lysed in 300 µL 8M urea with protease inhibitor (10% v/v lysate). After centrifugation (14,100g, 20 min), the supernatant was collected. Protein concentration was quantified via Bradford assay before storage at -80°C.

Protein Digestion & Desalting: Proteins (100 µg) were reduced (5 mM DTT, 37°C, 1h) and alkylated (10 mM iodoacetamide, RT, dark, 30min). Samples were diluted 4x with 25 mM ABC buffer, digested with trypsin (1:50 w/w, 37°C, overnight), and acidified with 0.1% FA. Digested peptides were desalted using C18 columns: washed with 0.1% FA and pH10 water, eluted with 70% ACN. Eluates were lyophilized and stored at -80°C.

LC-MS/MS Analysis: Peptides (500 ng) were separated on a 25-cm C18 column (150 μm ID, 1.9 µm beads) using an EASY nLC 1200 system with a 60-min gradient (8-40% B; Solvent A: 0.1% FA in H₂O, Solvent B: 80% ACN, 0.1% FA in H₂O) at 600 nL/min, 60°C. Analysis was performed on a Q Exactive HF-X mass spectrometer in data-dependent acquisition mode. Full MS scans (350-1500 m/z) were acquired at 120,000 resolution (AGC 3e6, max IT 80 ms). Top 40 precursors were isolated (1.6 m/z) and fragmented via HCD (NCE 27); MS/MS spectra were acquired at 15,000 resolution (AGC 5e4, max IT 45 ms) with 16s dynamic exclusion.

Protein ID/Quantitation: RAW files were processed in Proteome Discoverer 2.4 using Sequest HT against the human UniProtKB database. Search parameters: trypsin specificity (max 2 missed cleavages, min peptide length 6), fixed carbamidomethylation (Cys), variable oxidation (Met), ±15 ppm precursor mass tolerance, 0.02 Da fragment tolerance. PSMs and peptides were filtered to 1% FDR using Percolator. Proteins were inferred (1% FDR) with quantification based on unique/razor peptides.

Functional Analysis: Normalized data underwent missing value imputation (Perseus algorithm). Differentially expressed proteins (DEPs) were defined as average ratio-fold change >1.2 and p-value <0.05. DEP functional enrichment was assessed for GO terms (molecular function, biological process, cellular component), protein families (Pfam), pathways (KEGG, Reactome), and protein interactions (STRING-db). ROC analysis evaluated biomarker discriminative power. This work was performed by Beijing Qinglian Biotech Co., Ltd.

**mRNA Sequencing Methodology**

RNA Isolation and Library Preparation: Total RNA was extracted using TRIzol reagent following the manufacturer's protocol. RNA quality was assessed for purity/concentration (NanoDrop 2000) and integrity (Agilent 2100 Bioanalyzer). Sequencing libraries were constructed using the VAHTS Universal V10 RNA-seq Library Prep Kit^14, 15^. Library preparation and sequencing were performed by OE Biotech Co., Ltd. (Shanghai, China).

Sequencing and Read Processing: Libraries were sequenced on an Illumina Novaseq 6000 platform, generating 150 bp paired-end reads. Raw reads underwent quality control and adapter trimming using fastp, yielding clean reads for analysis.

Read Mapping and Quantification: Clean reads were aligned to the reference genome using HISAT2. Gene expression levels were quantified as FPKM using HTSeq-count. Principal Component Analysis (PCA) assessed sample reproducibility.

Differential Expression Analysis: Differentially Expressed Genes (DEGs) were identified using DESeq2 with significance thresholds: Q value < 0.05 and |log₂ fold-change| > 1 (fold-change > 2 or < 0.5). DEG expression patterns were visualized via hierarchical clustering and radar plots (top 30 genes) using R (v 3.2.0).

Functional Enrichment: Enriched terms/pathways for DEGs were identified using hypergeometric tests for GO, KEGG, Reactome, and WikiPathways^16, 17^. Results were visualized as column, chord, and bubble diagrams using R.

Gene Set Enrichment Analysis (GSEA): GSEA evaluated predefined gene sets for enrichment within the DEG expression ranking. This work was performed by OE Biotech Co., Ltd. (Shanghai, China).

**Cell Immunofluorescence**

Cells were washed, fixed in 4% paraformaldehyde for 10 min, and permeabilized. After blocking with donkey serum (30 min), primary antibodies were applied following tissue immunofluorescence protocols. Fluorophore-conjugated secondary antibodies were incubated for 60 min at room temperature, followed by DAPI nuclear staining and mounting.

**RNA Extraction**

Cell samples are lysed using a buffer, then mixed with a binding solution and loaded onto a purification column. Sequential washes are performed with two distinct wash buffers via centrifugation, followed by a high-speed centrifugation step to remove residual liquid. Pure RNA is eluted with elution buffer at room temperature and stored at -80°C or used for reverse transcription.

**Reverse Transcription**

Genomic DNA is eliminated in an RNase-free reaction mixture, followed by direct addition of reverse transcription reagents. The reaction proceeds at 37°C for 15 minutes and is inactivated at 85°C for 5 seconds.

**Quantitative PCR**

The PCR master mix contains SYBR Green dye, forward/reverse primers, and template. Amplification involves an initial denaturation at 95°C for 30 seconds, 40 cycles of 95°C for 15 seconds, 60°C for 15 seconds, and 72°C for 45 seconds (fluorescence data collection), concluding with a melting curve analysis.

**Soluble and insoluble ECM proteins (fibronectin and collagen) extraction**

This work was developed from existing protocol^18, 19^. Briefly, media was aspirated from the wells, and plates were placed on ice in an ice bucket. Cells were washed with 10 ml of ice-cold PBS, ensuring complete PBS removal; residual traces were eliminated by repeated washes. Subsequently, 300 μl of chilled deoxycholate lysis buffer containing inhibitors was added to each well, followed by immediate cell scraping. Cell lysates in lysis buffer were transferred to Eppendorf tubes, vortexed for 1 minute, and rotated on a rotating wheel in a cold room for 30 minutes (extending to 1 hour was acceptable). Tubes were centrifuged at 21,130g for 30 minutes at 4°C (if maximum speed was 15,871g, centrifugation time increased to 50 minutes).

For insoluble extracellular matrix proteins, 20 μl of chilled SDS lysis buffer with inhibitors was added to the pellet and mixed by pipetting. The pellet was heated at 95°C for 1 minute until fully dissolved, labeled as "insoluble fraction," mixed with 5 μl of 5× SDS loading dye, and stored at −20°C. For soluble proteins, 200 μl of supernatant was transferred to a new tube, labeled "soluble fraction," combined with 50 μl of 5× SDS loading dye, and stored at −20°C. Before loading, both "soluble" and "insoluble" samples were heated at 95°C for 5 minutes and centrifuged at 21,130g for 1 minute at room temperature.

**Enzyme linked immunosorbent assay (ELISA)**

Standards and diluted test samples were loaded into designated wells, followed by the addition of enzyme-conjugated reagent (excluding blank wells) and incubation at 37°C for 60 minutes. After five washes and plate drying, substrates A and B were added sequentially for 15-minute color development in the dark. The reaction was terminated with stop solution, and absorbance (OD) at 450 nm was measured within 15 minutes.

**Molecular Modeling, Docking, and Visualization**

The three-dimensional structure of the protein of interest was predicted using AlphaFold 3 (version 3.0.0) The canonical amino acid sequence of human fibronectin was retrieved from the Protein Data Bank to serve as the primary template. For the peptides pUR4 and R1R2, their sequences were as described in the Methods section. Among all generated models, the one with the highest predicted local distance difference test (pLDDT) score was selected for subsequent analysis. Molecular docking was then performed using AutoDock Vina (version 1.2.5) to investigate potential binding modes and affinities. The protein structure was prepared by adding polar hydrogens and assigning Gasteiger charges. The docking grid was centered on the putative binding site with dimensions sufficient to accommodate the ligand. The default search parameters were used, and the docking pose with the most favorable binding affinity (lowest Gibbs free energy, ΔG) was selected for further analysis. Structural visualization, analysis of the predicted model and docking complexes, and generation of publication-quality figures were performed using PyMOL Molecular Graphics System (version 2.5.0)

Here's the amino acid sequence in detail.

pUR4:

KDQSPLAGESGETEYITEVYGNQQNPVDIDKKLPNETGFSGNMVETEDT

R1R2: GLNGENQKEPEQGERGEAGPPLSGLSGNNQGRPSLPGLNGENQKEPEQGERGEAGPP

Fibronectin: MLRGPGPGLLLLAVQCLGTAVPSTGASKSKRQAQQMVQPQSPVAVSQSKPGCYDNGKHYQINQQWERTYLGNALVCTCYGGSRGFNCESKPEAEETCFDKYTGNTYRVGDTYERPKDSMIWDCTCIGAGRGRISCTIANRCHEGGQSYKIGDTWRRPHETGGYMLECVCLGNGKGEWTCKPIAEKCFDHAAGTSYVVGETWEKPYQGWMMVDCTCLGEGSGRITCTSRNRCNDQDTRTSYRIGDTWSKKDNRGNLLQCICTGNGRGEWKCERHTSVQTTSSGSGPFTDVRAAVYQPQPHPQPPPYGHCVTDSGVVYSVGMQWLKTQGNKQMLCTCLGNGVSCQETAVTQTYGGNSNGEPCVLPFTYNGRTFYSCTTEGRQDGHLWCSTTSNYEQDQKYSFCTDHTVLVQTRGGNSNGALCHFPFLYNNHNYTDCTSEGRRDNMKWCGTTQNYDADQKFGFCPMAAHEEICTTNEGVMYRIGDQWDKQHDMGHMMRCTCVGNGRGEWTCIAYSQLRDQCIVDDITYNVNDTFHKRHEEGHMLNCTCFGQGRGRWKCDPVDQCQDSETGTFYQIGDSWEKYVHGVRYQCYCYGRGIGEWHCQPLQTYPSSSGPVEVFITETPSQPNSHPIQWNAPQPSHISKYILRWRPKNSVGRWKEATIPGHLNSYTIKGLKPGVVYEGQLISIQQYGHQEVTRFDFTTTSTSTPVTSNTVTGETTPFSPLVATSESVTEITASSFVVSWVSASDTVSGFRVEYELSEEGDEPQYLDLPSTATSVNIPDLLPGRKYIVNVYQISEDGEQSLILSTSQTTAPDAPPDTTVDQVDDTSIVVRWSRPQAPITGYRIVYSPSVEGSSTELNLPETANSVTLSDLQPGVQYNITIYAVEENQESTPVVIQQETTGTPRSDTVPSPRDLQFVEVTDVKVTIMWTPPESAVTGYRVDVIPVNLPGEHGQRLPISRNTFAEVTGLSPGVTYYFKVFAVSHGRESKPLTAQQTTKLDAPTNLQFVNETDSTVLVRWTPPRAQITGYRLTVGLTRRGQPRQYNVGPSVSKYPLRNLQPASEYTVSLVAIKGNQESPKATGVFTTLQPGSSIPPYNTEVTETTIVITWTPAPRIGFKLGVRPSQGGEAPREVTSDSGSIVVSGLTPGVEYVYTIQVLRDGQERDAPIVNKVVTPLSPPTNLHLEANPDTGVLTVSWERSTTPDITGYRITTTPTNGQQGNSLEEVVHADQSSCTFDNLSPGLEYNVSVYTVKDDKESVPISDTIIPEVPQLTDLSFVDITDSSIGLRWTPLNSSTIIGYRITVVAAGEGIPIFEDFVDSSVGYYTVTGLEPGIDYDISVITLINGGESAPTTLTQQTAVPPPTDLRFTNIGPDTMRVTWAPPPSIDLTNFLVRYSPVKNEEDVAELSISPSDNAVVLTNLLPGTEYVVSVSSVYEQHESTPLRGRQKTGLDSPTGIDFSDITANSFTVHWIAPRATITGYRIRHHPEHFSGRPREDRVPHSRNSITLTNLTPGTEYVVSIVALNGREESPLLIGQQSTVSDVPRDLEVVAATPTSLLISWDAPAVTVRYYRITYGETGGNSPVQEFTVPGSKSTATISGLKPGVDYTITVYAVTGRGDSPASSKPISINYRTEIDKPSQMQVTDVQDNSISVKWLPSSSPVTGYRVTTTPKNGPGPTKTKTAGPDQTEMTIEGLQPTVEYVVSVYAQNPSGESQPLVQTAVTNIDRPKGLAFTDVDVDSIKIAWESPQGQVSRYRVTYSSPEDGIHELFPAPDGEEDTAELQGLRPGSEYTVSVVALHDDMESQPLIGTQSTAIPAPTDLKFTQVTPTSLSAQWTPPNVQLTGYRVRVTPKEKTGPMKEINLAPDSSSVVVSGLMVATKYEVSVYALKDTLTSRPAQGVVTTLENVSPPRRARVTDATETTITISWRTKTETITGFQVDAVPANGQTPIQRTIKPDVRSYTITGLQPGTDYKIYLYTLNDNARSSPVVIDASTAIDAPSNLRFLATTPNSLLVSWQPPRARITGYIIKYEKPGSPPREVVPRPRPGVTEATITGLEPGTEYTIYVIALKNNQKSEPLIGRKKTDELPQLVTLPHPNLHGPEILDVPSTVQKTPFVTHPGYDTGNGIQLPGTSGQQPSVGQQMIFEEHGFRRTTPPTTATPIRHRPRPYPPNVGEEIQIGHIPREDVDYHLYPHGPGLNPNASTGQEALSQTTISWAPFQDTSEYIISCHPVGTDEEPLQFRVPGTSTSATLTGLTRGATYNVIVEALKDQQRHKVREEVVTVGNSVNEGLNQPTDDSCFDPYTVSHYAVGDEWERMSESGFKLLCQCLGFGSGHFRCDSSRWCHDNGVNYKIGEKWDRQGENGQMMSCTCLGNGKGEFKCDPHEATCYDDGKTYHVGEQWQKEYLGAICSCTCFGGQRGWRCDNCRRPGGEPSPEGTTGQSYNQYSQRYHQRTNTNVNCPIECFMPLDVQADREDSRE

1. Singh P, Carraher C, Schwarzbauer JE. Assembly of fibronectin extracellular matrix. Annu Rev Cell Dev Biol 2010;26:397-419.

2. Ensenberger MG, Tomasini-Johansson BR, Sottile J, et al. Specific interactions between F1 adhesin of Streptococcus pyogenes and N-terminal modules of fibronectin. J Biol Chem 2001;276:35606-13.

3. Atkin KE, Brentnall AS, Harris G, et al. The streptococcal binding site in the gelatin-binding domain of fibronectin is consistent with a non-linear arrangement of modules. J Biol Chem 2010;285:36977-83.

4. Harris G, Ma W, Maurer LM, et al. Borrelia burgdorferi protein BBK32 binds to soluble fibronectin via the N-terminal 70-kDa region, causing fibronectin to undergo conformational extension. J Biol Chem 2014;289:22490-9.

5. Hatley RJD, Macdonald SJF, Slack RJ, et al. An αv-RGD Integrin Inhibitor Toolbox: Drug Discovery Insight, Challenges and Opportunities. Angew Chem Int Ed Engl 2018;57:3298-3321.

6. Slack RJ, Macdonald SJF, Roper JA, et al. Emerging therapeutic opportunities for integrin inhibitors. Nat Rev Drug Discov 2022;21:60-78.

7. Danese S, Sans M, Spencer DM, et al. Angiogenesis blockade as a new therapeutic approach to experimental colitis. Gut 2007;56:855-62.

8. Zhilan T, Zengyu Z, Pengpeng J, et al. Salidroside promotes pro-angiogenesis and repair of blood brain barrier via Notch/ITGB1 signal path in CSVD Model. J Adv Res 2025;68:429-444.

9. Khalili P, Arakelian A, Chen G, et al. A non-RGD-based integrin binding peptide (ATN-161) blocks breast cancer growth and metastasis in vivo. Mol Cancer Ther 2006;5:2271-80.

10. Stoeltzing O, Liu W, Reinmuth N, et al. Inhibition of integrin alpha5beta1 function with a small peptide (ATN-161) plus continuous 5-FU infusion reduces colorectal liver metastases and improves survival in mice. Int J Cancer 2003;104:496-503.

11. Lou Y, Sun H, Morrissey S, et al. Critical roles of TIPE2 protein in murine experimental colitis. J Immunol 2014;193:1064-70.

12. Wu X, Li X, Song Y, et al. Allicin protects auditory hair cells and spiral ganglion neurons from cisplatin - Induced apoptosis. Neuropharmacology 2017;116:429-440.

13. Li Z, Yi X, Zhou H, et al. Combined effect of polystyrene microplastics and dibutyl phthalate on the microalgae Chlorella pyrenoidosa. Environ Pollut 2020;257:113604.

14. Chen S, Zhou Y, Chen Y, et al. fastp: an ultra-fast all-in-one FASTQ preprocessor. Bioinformatics 2018;34:i884-i890.

15. Roberts A, Trapnell C, Donaghey J, et al. Improving RNA-Seq expression estimates by correcting for fragment bias. Genome Biol 2011;12:R22.

16. The Gene Ontology Resource: 20 years and still GOing strong. Nucleic Acids Res 2019;47:D330-d338.

17. Kanehisa M, Araki M, Goto S, et al. KEGG for linking genomes to life and the environment. Nucleic Acids Res 2008;36:D480-4.

18. Varadaraj A, Magdaleno C, Mythreye K. Deoxycholate Fractionation of Fibronectin (FN) and Biotinylation Assay to Measure Recycled FN Fibrils in Epithelial Cells. Bio Protoc 2018;8.

19. Keski-Oja J, Todaro GJ. Specific effects of fibronectin-releasing peptides on the extracellular matrices of cultured human fibroblasts. Cancer Res 1980;40:4722-7.

**Supplementary figures**

**
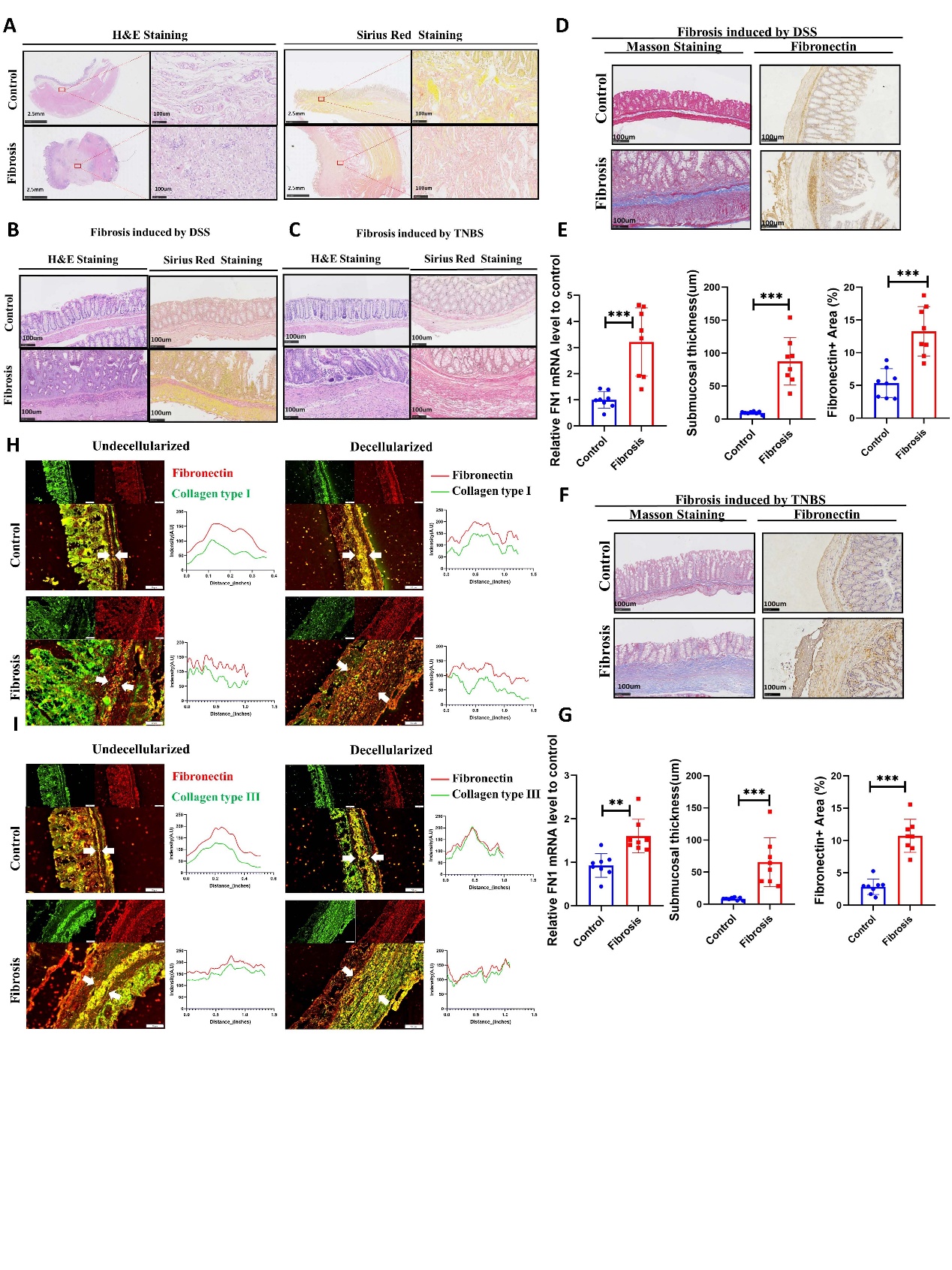
**

**Figure S1. Fibrosis predominantly localizes to the intestinal submucosa.**

(A) Representative H&E and Sirius red-stained sections of healthy (n=10) and fibrotic (n=10) intestinal tissues from human clinical specimens.

(B) Representative H&E and Sirius red-stained sections of healthy (n=8) and fibrotic (n=8) intestinal tissues in the DSS-induced intestinal fibrosis model.

(C) Representative H&E and Sirius red-stained sections of healthy (n=8) and fibrotic (n=8) intestinal tissues in the TNBS-induced intestinal fibrosis model. Obvious submucosal thickening with pink (H&E) or red (Sirius red) fibrotic extracellular matrix was observed in all fibrotic samples.

(D) Representative Masson’s trichrome and fibronectin IHC of healthy (Control group, n=8) and fibrotic (Fibrosis group, n=8) intestines in the DSS-induced intestinal fibrosis model.

(E) Quantification of submucosal thickness and fibronectin abundance in the DSS model.

(F) Representative Masson’s trichrome and fibronectin IHC of healthy (Control group, n=8) and fibrotic (Fibrosis group, n=8) intestines in the TNBS-induced intestinal fibrosis model. Different from the DSS model, TNBS-induced fibrosis features submucosal thickening and muscularis propria damage, with dense ECM and fibronectin accumulation in both regions.

(G) Quantification of submucosal thickness and fibronectin abundance in the TNBS model.

(H, I) Representative images and colocalization analysis of fibronectin with type I and III collagen in native and decellularized tissues under healthy and fibrotic conditions.


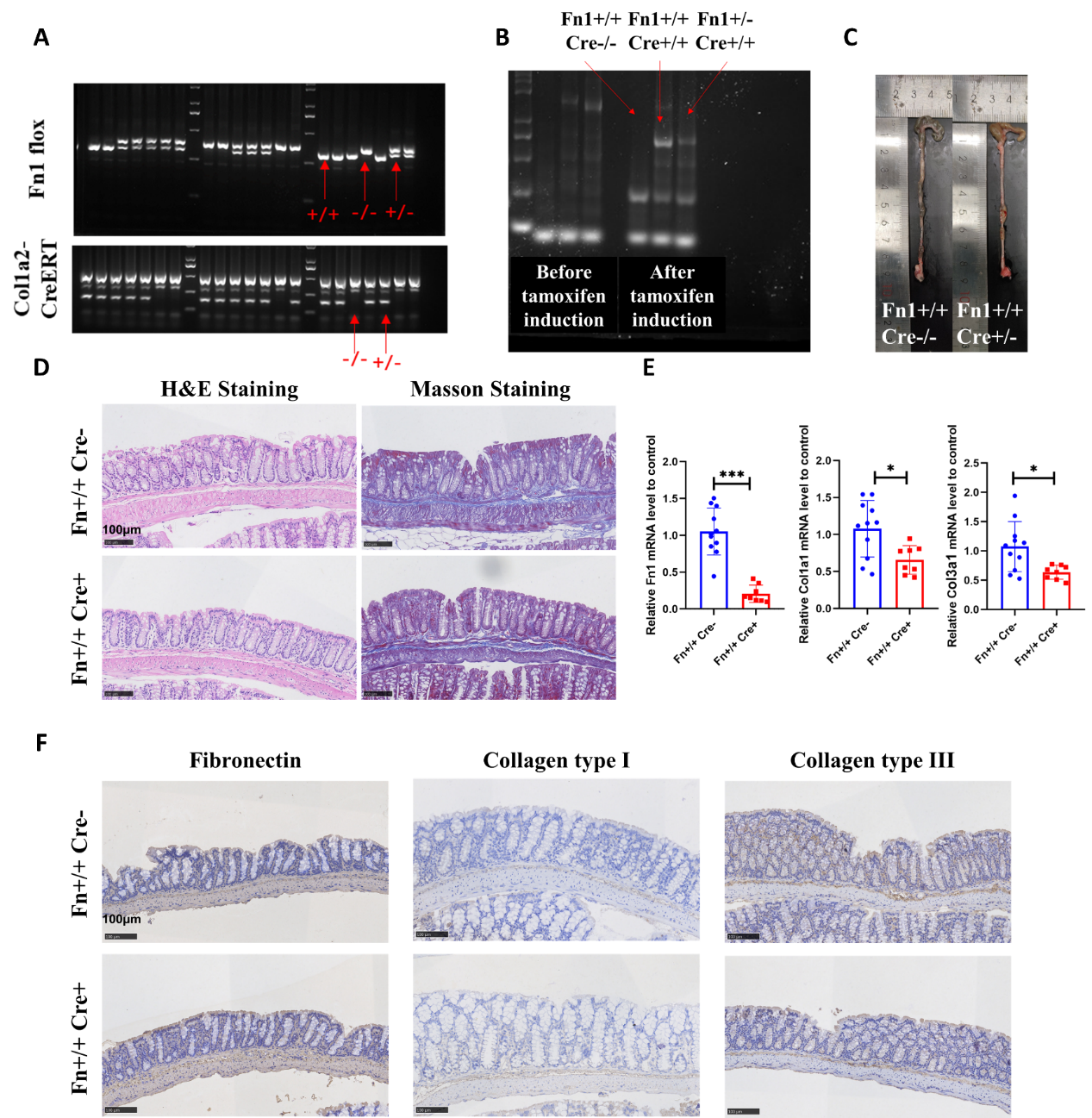


**Figure S2. Construction and baseline characterization of Fn1 conditional knockout (cKO) mice.**

(A) Genotyping results for Fn1-Flox and Col1a2-CreERT-Tg mice.

(B) Genotyping of cKO mice post-tamoxifen induction.

(C) Gross specimens of cKO mouse intestines at baseline; no significant differences in intestinal length were observed.

(D) Representative H&E and Masson’s trichrome staining of cKO intestines at baseline; Fn1 deletion did not alter intestinal architecture.

(E) Transcript levels of Fn1, Col1a1, and Col3a1 in baseline cKO mice. Fn1 knockout significantly reduced fibronectin, collagen I, and collagen III transcription.

(F) Representative IHC staining for fibronectin, collagen I, and collagen III in baseline cKO mice; no significant changes in main ECM proteins were detected. Groups: Fn+/+ Cre-, n=11; Fn+/+ Cre+, n=8. Data are presented as mean ±SD. *, p<0.05; **, p<0.01; ***, p<0.001. IHC, immunohistochemistry; ECM, extracellular matrix.


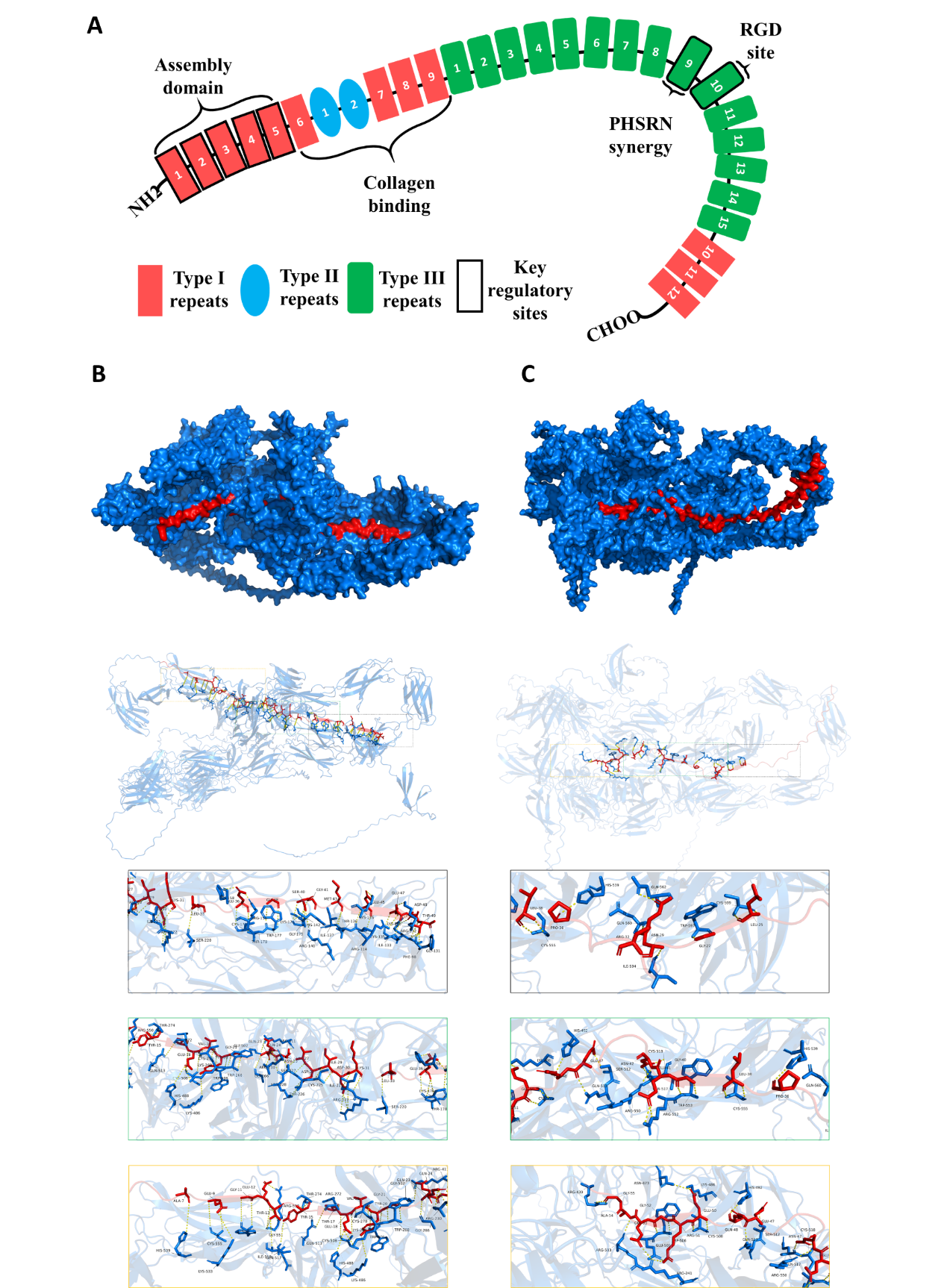


**Figure S3. Schematics of fibronectin and inhibitor docking.**

(A) Domain architecture of fibronectin: Type I repeats (red rectangles), Type II repeats (blue ovals), Type III repeats (green rounded rectangles); assembly-regulating sites (black borders). Detailed in the Supplement.

(B) Molecular docking of pUR4 with fibronectin. Insets show magnified regions (black, green, yellow dashed boxes). Detailed information regarding amino acid tags can be found in Fig S4

(C) Molecular docking of R1R2 with fibronectin. Insets show magnified regions (conventions as in B). Detailed information regarding amino acid tags can be found in Fig S5


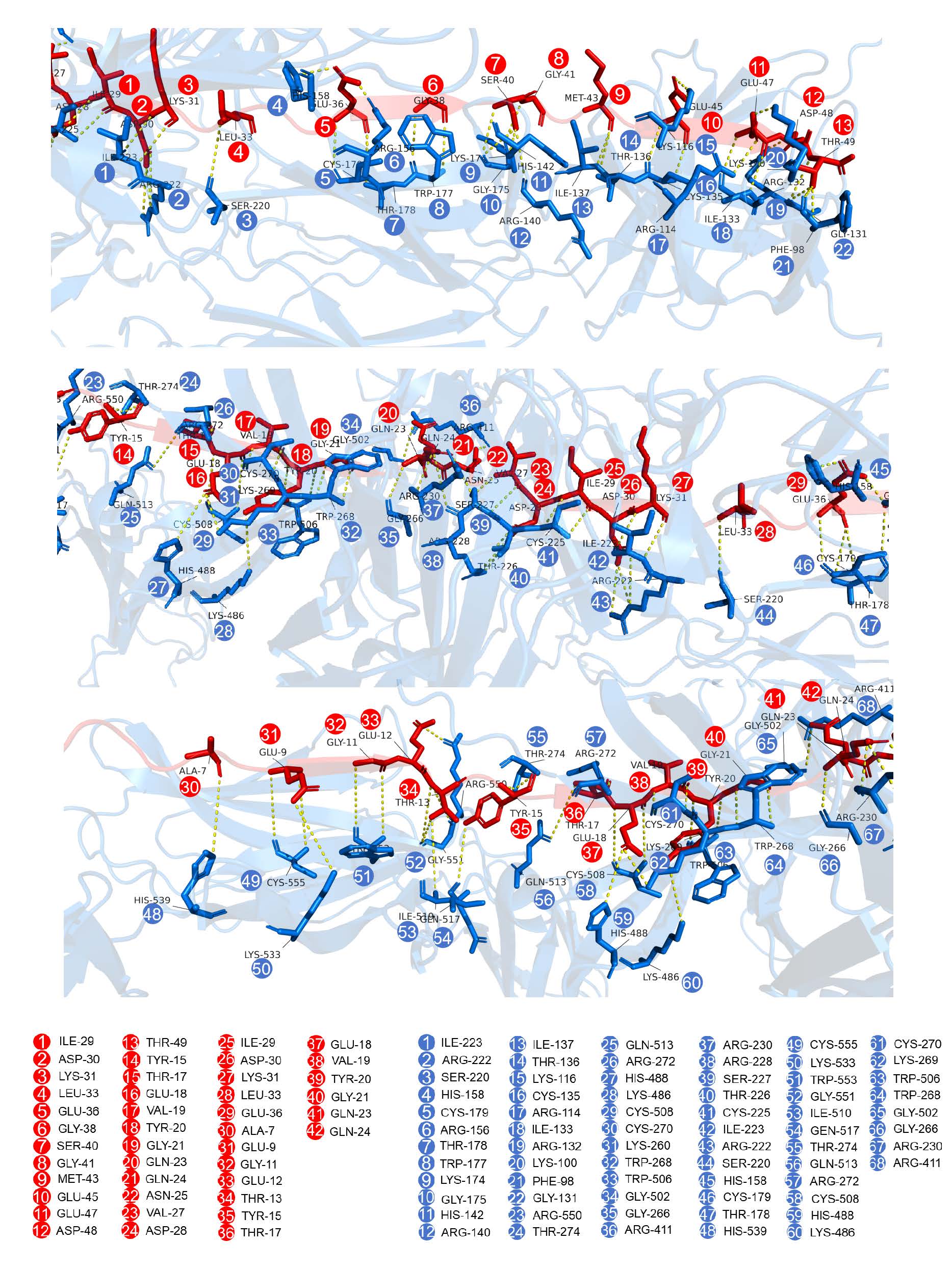


**Figure S4. Detailed amino acid interaction display of pUR4 and fibronectin molecular docking**

**
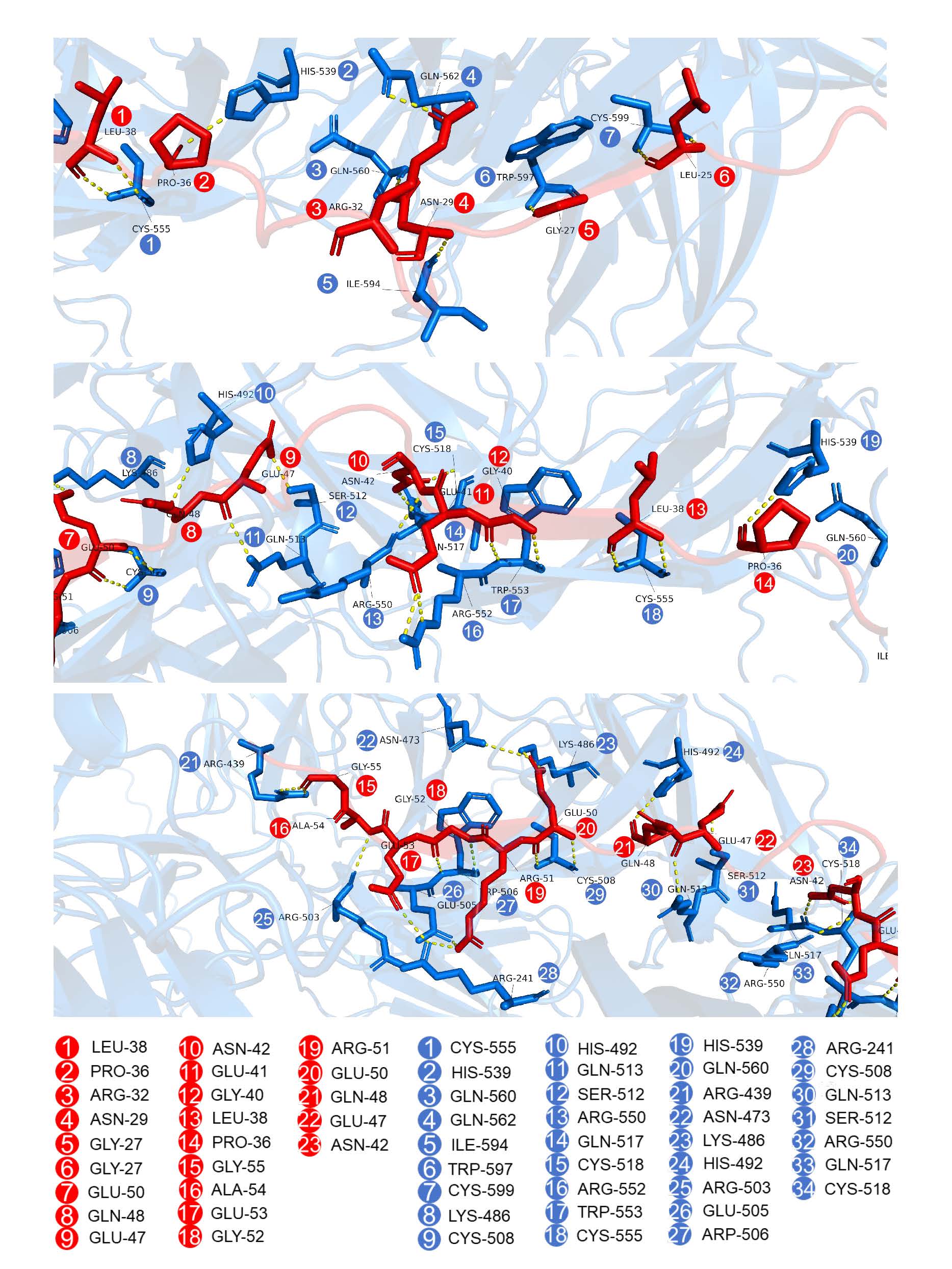
**

**Figure S5. Detailed amino acid interaction display of R1R2 and fibronectin molecular docking**


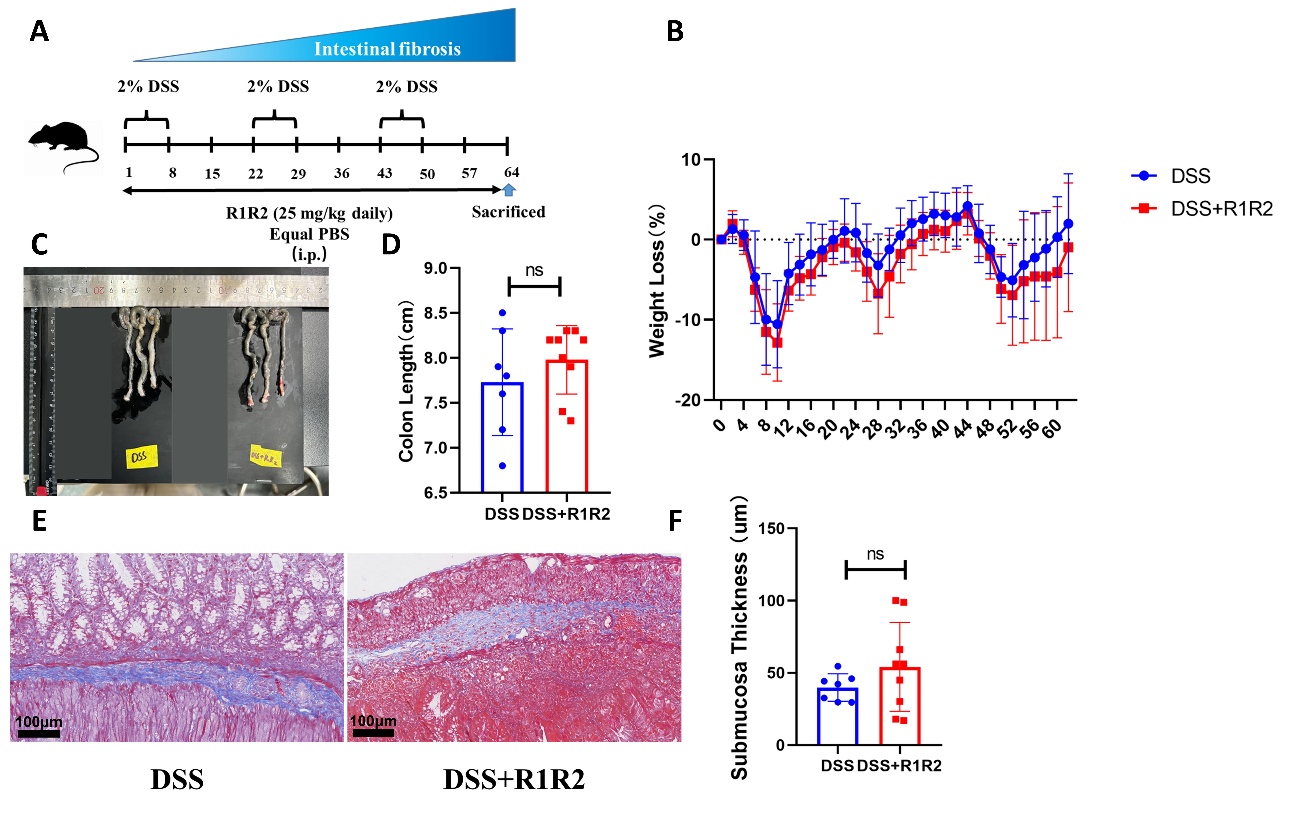


**Figure S6. Suboptimal amelioration of intestinal fibrosis by R1R2.**

(A) Dosing protocol for DSS model: Mice received daily i.p. R1R2 (25 mg/kg) or PBS from DSS initiation until endpoint (DSS, n=7; DSS+R1R2, n=9).

(B) Body weight trends (no significant difference between groups).

(C, D) Macroscopic colon views and length quantification.

(E) Representative Masson’s trichrome staining.

(F) Submucosal thickness quantification. Data are presented as mean ±SD. *, p<0.05; **, p<0.01; ***, p<0.001. DSS, dextran sulfate sodium salt.


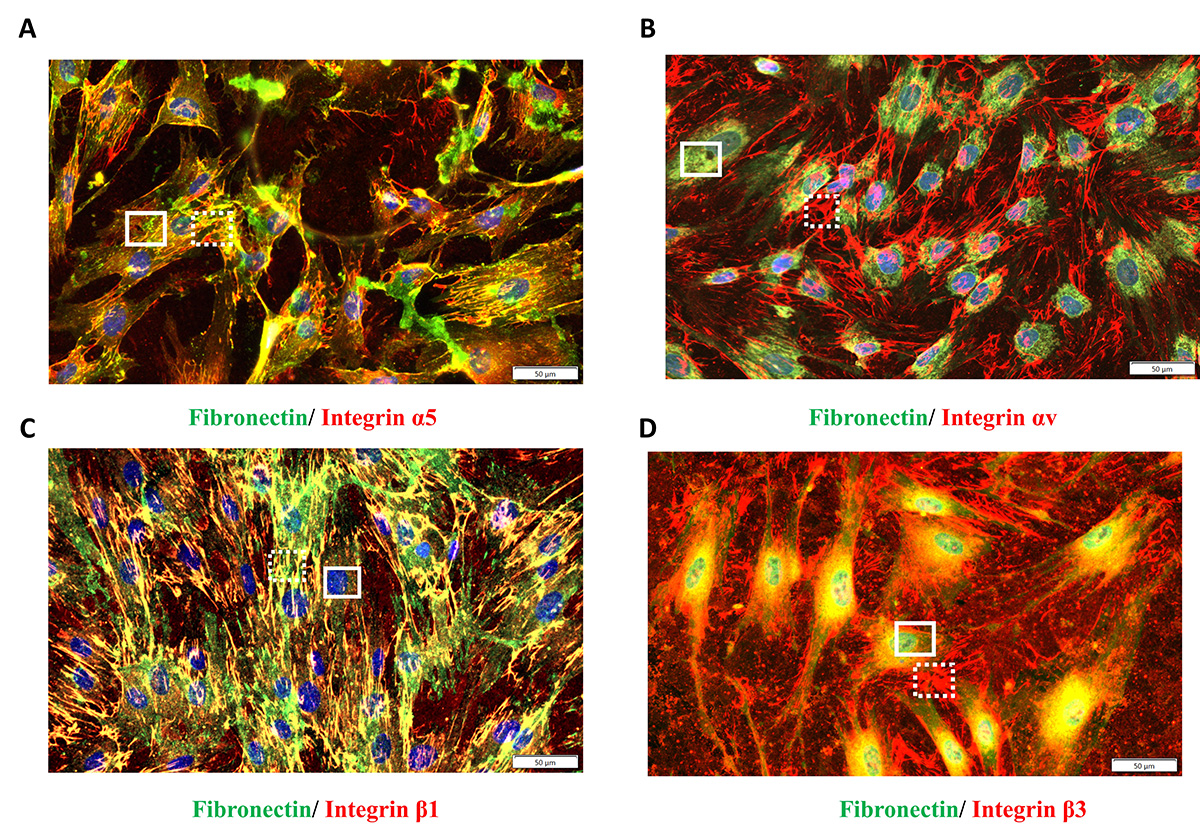


**Figure S7.** **Display of selected parts for co-localization analysis.**

1. Display of selected parts for co-localization analysis of fibronectin and integrin α5

(B) Display of selected parts for co-localization analysis of fibronectin and integrin αv

(C) Display of selected parts for co-localization analysis of fibronectin and integrin β1

(D) Display of selected parts for co-localization analysis of fibronectin and integrin β3

The solid line box shows the region used for intracellular co-localization analysis. The dotted line box shows the region used for extracellular matrix co-localization analysis.


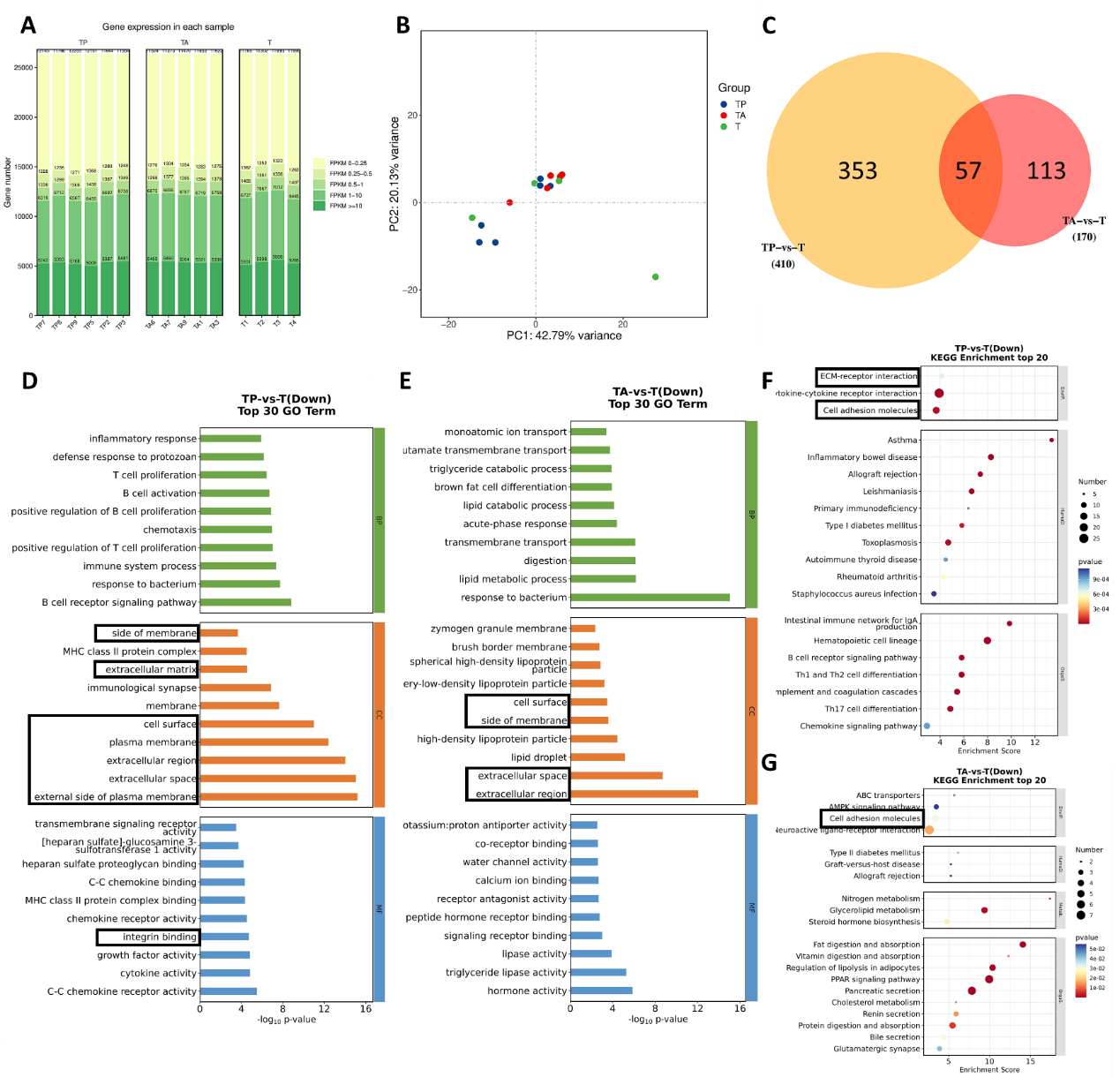


**Figure S8.** **RNA Sequencing of Fibronectin-Inhibitor Treatment in Intestinal Fibrosis**

(A) Expression distribution differences of sequenced genes. (B) Principal component analysis (PCA). (C) Venn diagram of differentially expressed genes (DEGs). (D) GO enrichment of DEGs induced by pUR4 intervention. (E) GO enrichment of DEGs induced by ATN161 intervention. (F) KEGG enrichment of DEGs induced by pUR4 intervention. (G) KEGG enrichment of DEGs induced by ATN161 intervention. Black boxes highlight enrichments related to extracellular matrix accumulation. GO, Gene Ontology; KEGG, Kyoto Encyclopedia of Genes and Genomes.


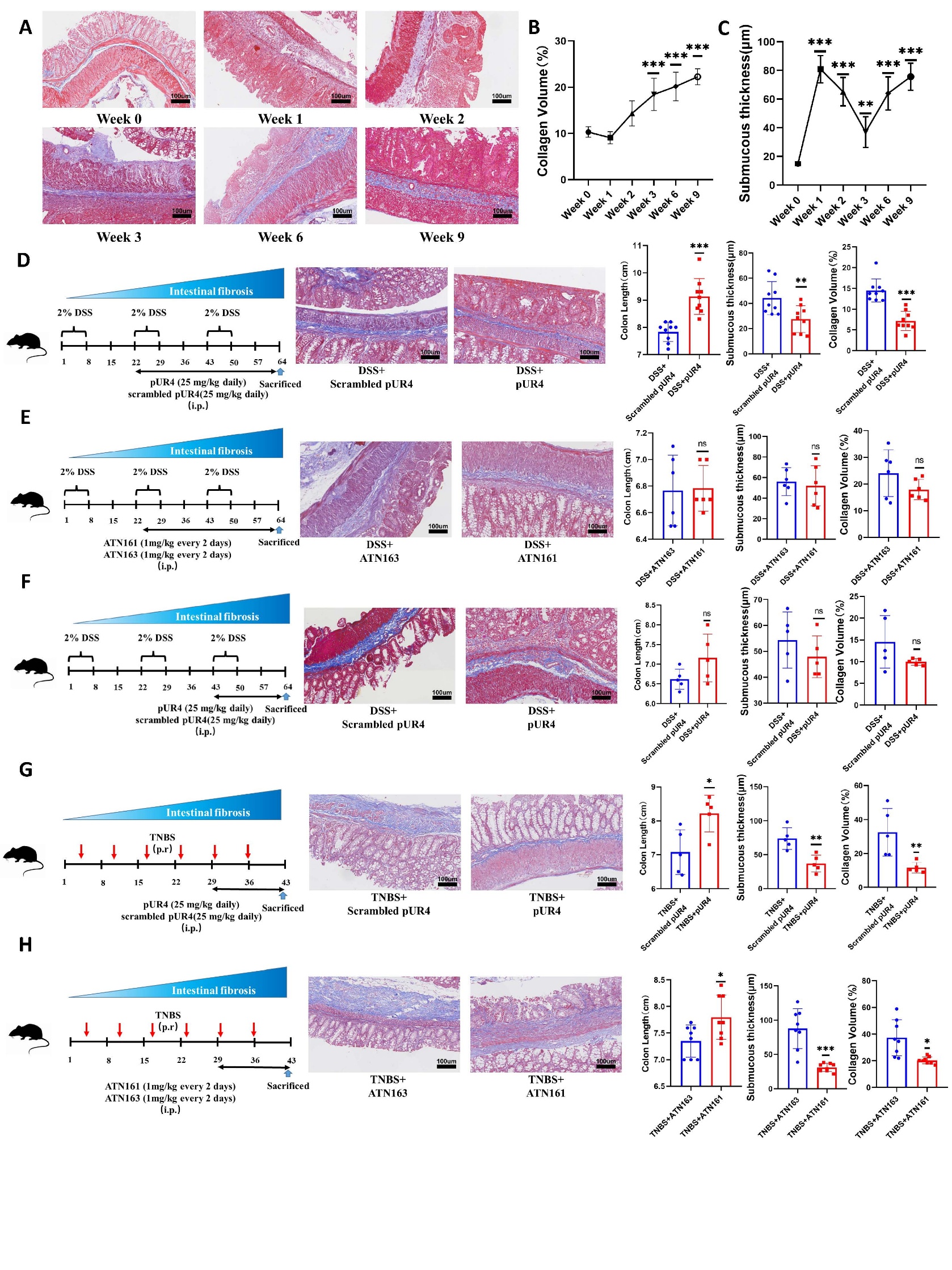


**Figure S9.** **Inhibition of Fibronectin Ameliorates Established Intestinal Fibrosis**

(A-C) Representative Masson staining images (A), collagen volume changes (B), and submucosal changes (C) in the DSS model as the modeling period progressed (n=5 for each group).

(D) Effect of pUR4 treatment initiated in the fourth week on the DSS model. (DSS + scrambled pUR4: n=9; DSS + pUR4: n=9)

(E) Effect of ATN161 treatment initiated in the fourth week on the DSS model. (DSS + ATN163: n=6; DSS +ATN161: n=6)

(F) Effect of pUR4 treatment initiated in the seventh week on the DSS model. (DSS + scrambled pUR4: n=5; DSS + pUR4: n=5)

(G) Effect of pUR4 treatment initiated in the fifth week on the TNBS model. (TNBS + scrambled pUR4: n=5; TNBS + pUR4: n=5)

(H) Effect of ATN161 treatment initiated in the fifth week on the TNBS model. (TNBS + ATN163: n=8; TNBS +ATN161: n=8)

Data are presented as mean ±SD. *, p<0.05; **, p<0.01; ***, p<0.001. Continuous data were analyzed using Student's t-test or ANOVA for unpaired groups. Nonparametric distributions were analyzed by Mann-Whitney/Wilcoxon rank-sum tests

Table S1 Antibody information

| Antibody | Host | Dilution | | | Antigen retrieval | Company | Cat No. |
| --- | --- | --- | --- | --- | --- | --- | --- |
|  |  | IHC | IF | WB |  |  |  |
| Integrin alpha 5 | Rabbit | 1:500 | 1:300 | N/A | Tris/EDTA buffer pH 9.0 | abcam | ab150361 |
| Integrin beta 1 | Rabbit | 1:1000 | 1:300 | N/A | Tris/EDTA buffer pH 9.0 | abcam | ab179471 |
| Integrin alpha V | Rabbit | 1:500 | 1:300 | N/A | Tris/EDTA buffer pH 9.0 | abcam | ab179475 |
| Integrin beta 3 | Rabbit | 1:500 | 1:300 | N/A | Tris/EDTA buffer pH 9.0 | abcam | ab179473 |
| Fibronectin | Goat | 1:2000 | 1:1000 | N/A | Tris/EDTA buffer pH 9.0 | manufacture | N/A |
| Fibronectin | Rabbit | 1:400 | 1:200 | N/A | Tris/EDTA buffer pH 9.0 | CST | #26836 |
| Fibronectin | Mouse | N/A | 1:400 | 1:10000 | Tris/EDTA buffer pH 9.0 | proteintech | 66042-1-Ig |
| Collagen I | Rabbit | N/A | 1:3000 | N/A | Tris/EDTA buffer pH 9.0 | proteintech | 86093-1-RR |
| Collagen I | Mouse | 1:2000 | 1:600 | 1:20000 | Tris/EDTA buffer pH 9.0 | proteintech | 67288-1-Ig |
| Collagen III | Rabbit | 1:2000 | 1:500 | N/A | Tris/EDTA buffer pH 9.0 | proteintech | 22734-1-AP |
| α SMA | Rabbit | N/A | 1:500 | N/A | Tris/EDTA buffer pH 9.0 | proteintech | 14395-1-AP |
| β actin | Mouse | N/A | NA/ | 1:20000 | N/A | proteintech | 66009-1-Ig |
| Dylight 488, Goat Anti-Rabbit IgG | Goat | N/A | 1:1000 | N/A | N/A | Abbkine | A23220 |
| Dylight 594, Goat Anti-Rabbit IgG | Goat | N/A | 1:1000 | N/A | N/A | Abbkine | A23420 |
| DyLight 488, Goat Anti-Mouse IgG | Goat | N/A | 1:1000 | N/A | N/A | Abbkine | A23210 |
| Dylight 594, Goat Anti-Mouse IgG | Goat | N/A | 1:1000 | N/A | N/A | Abbkine | A23410 |
| IFKine™ Green Donkey Anti-Goat IgG | Donkey | N/A | 1:1000 | N/A | N/A | Abbkine | A24231 |

Table S2 Primer sequences for qPT-PCR

| Gene | Species | Forward primer | Reverse primer |
| --- | --- | --- | --- |
| ITGAV | Human | AGGAGAAGGTGCCTACGAAGCT | GCACAGGAAAGTCTTGCTAAGGC |
| ITGB1 | Human | GGATTCTCCAGAAGGTGGTTTCG | TGCCACCAAGTTTCCCATCTCC |
| ITGA5 | Human | GCCGATTCACATCGCTCTCAAC | GTCTTCTCCACAGTCCAGCAAG |
| ITGB3 | Human | CATGGATTCCAGCAATGTCCTCC | TTGAGGCAGGTGGCATTGAAGG |
| ACTA2 | Human | CTATGCCTCTGGACGCACAACT | CAGATCCAGACGCATGATGGCA |
| FN1 | Human | ACAACACCGAGGTGACTGAGAC | GGACACAACGATGCTTCCTGAG |
| COL1A1 | Human | GATTCCCTGGACCTAAAGGTGC | AGCCTCTCCATCTTTGCCAGCA |
| COL1A2 | Human | CCTGGTGCTAAAGGAGAAAGAGG | ATCACCACGACTTCCAGCAGGA |
| COL3A1 | Human | TGGTCTGCAAGGAATGCCTGGA | TCTTTCCCTGGGACACCATCAG |
| RHOA | Human | TCTGTCCCAACGTGCCCATCAT | CTGCCTTCTTCAGGTTTCACCG |
| ROCK2 | Human | TGCGGTCACAACTCCAAGCCTT | CGTACAGGCAATGAAAGCCATCC |
| ROCK1 | Human | GAAACAGTGTTCCATGCTAGACG | GCCGCTTATTTGATTCCTGCTCC |
| CFL1 | Human | GCAACCTATGAGACCAAGGAGAG | TCTTGATGGCGTCCTTGGAGCT |
| PTK2 | Human | GCCTTATGACGAAATGCTGGGC | CCTGTCTTCTGGACTCCATCCT |
| SRC | Human | CTGCTTTGGCGAGGTGTGGATG | CCACAGCATACAACTGCACCAG |
| PXN | Human | CTGATGGCTTCGCTGTCGGATT | GCTTGTTCAGGTCAGACTGCAG |
| TSN1 | Human | ACTCCAGAGGAGGAGCCATTGA | TGTGGCTTCTGGAGACTGGTTC |
| GAPDH | Human | GTCTCCTCTGACTTCAACAGCG | ACCACCCTGTTGCTGTAGCCAA |
| Gapdh | Mouse | CATCACTGCCACCCAGAAGACTG | ATGCCAGTGAGCTTCCCGTTCAG |
| Fn1 | Mouse | CCCTATCTCTGATACCGTTGTCC | TGCCGCAACTACTGTGATTCGG |
| Cola1 | Mouse | CCTCAGGGTATTGCTGGACAAC | CAGAAGGACCTTGTTTGCCAGG |
| Col3a1 | Mouse | GACCAAAAGGTGATGCTGGACAG | CAAGACCTCGTGCTCCAGTTAG |
| Fn1-flox P1 | Mouse | GTCCCGAAAGCCTCCATGAT | |
| Fn1-flox P2 | Mouse | TCTCGGGAAATGTGGGCAAA | |
| Fn1-flox P3 (Cre test) | Mouse | GTCCCGAAAGCCTCCATGAT | |
| Fn1-flox P4 (Cre test) | Mouse | TTCGACCGAGTGAGTTGACG | |
| Col1a2-CreERT 1 | Mouse | CATGTCCATCAGGTTCTTGC | |
| Col1a2-CreERT 2 | Mouse | CAGGAGGTTTCGACTAAGTTGG | |
| Col1a2-CreERT 3 | Mouse | AGTGGCCTCTTCCAGAAATG | |
| Col1a2-CreERT 4 | Mouse | TGCGACTGTGTCTGATTTCC | |

Table S3 Clinical Characteristics of CD Patients with Intestinal Stenosis.

| Patient ID | Gender | Age | Stricture site | intestinal perforation |
| --- | --- | --- | --- | --- |
| 1 | Male | 42 | ileum | No |
| 2 | Male | 34 | ileum | Yes |
| 3 | Female | 21 | ileum | Yes |
| 4 | Male | 29 | ileum | No |
| 5 | Female | 57 | ileum | Yes |
| 6 | Male | 22 | ileum and Ileocecal junction | No |
| 7 | Male | 33 | ileum, Ileocecal junction and colon | Yes |
| 8 | Male | 43 | ileum | No |
| 9 | Male | 27 | ileum | Yes |
| 10 | Male | 20 | ileocecal junction and colon | No |

Table S4 Matrisome Analysis Results of Major Extracellular Matrix Components

| Accession | Gene Name | Proportion （%） | | | |
| --- | --- | --- | --- | --- | --- |
|  |  | Conrol | Decellularized control | Decellularized fibrosis | Fibrosis |
| Q60847 | Col12a1 | 7.738678 | 4.924489 | 8.211007 | 10.40835 |
| Q80X19 | Col14a1 | 2.229293 | 0.956872 | 1.727446 | 3.82484 |
| O35206 | Col15a1 | 0.825854 | 0.631322 | 0.530048 | 0.883603 |
| P39061 | Col18a1 | 1.173171 | 0.485237 | 0.868766 | 1.863323 |
| P11087 | Col1a1 | 4.737583 | 6.19356 | 4.887312 | 3.506746 |
| Q01149 | Col1a2 | 2.812393 | 4.818901 | 3.382481 | 2.209572 |
| P08121 | Col3a1 | 0.458474 | 0.488614 | 0.345216 | 0.448512 |
| P02463 | Col4a1 | 1.066091 | 0.774404 | 0.716657 | 0.87934 |
| P08122 | Col4a2 | 1.970591 | 2.623536 | 1.678957 | 1.247591 |
| O88207 | Col5a1 | 0.269327 | 0.238268 | 0.207677 | 0.115859 |
| Q3U962 | Col5a2 | 0.694545 | 0.429629 | 0.25453 | 0.505209 |
| Q9JLI2 | Col5a3 | 0.167806 | 0.10704 | 0.07385 | 0.1523 |
| Q04857 | Col6a1 | 5.970197 | 11.28747 | 7.847148 | 5.317744 |
| Q02788 | Col6a2 | 7.975214 | 9.486836 | 6.052771 | 6.844125 |
| A0A087WS16 | Col6a3 | 18.70734 | 27.09716 | 16.96841 | 14.73291 |
| E9PWQ3 | Col6a3 | 0.0401 | 0.130832 | 0.155249 | 0.088027 |
| A2AX52 | Col6a4 | 0.210006 | 1.172585 | 0.840316 | 0.237019 |
| A6H584 | Col6a5 | 0.412626 | 0.791898 | 0.705484 | 0.331239 |
| P54320 | Eln | 0.154254 | 0.181591 | 0.204206 | 0.08584 |
| Q61554 | Fbn1 | 3.3913 | 3.389886 | 3.26329 | 2.355643 |
| A0A087WR50 | Fn1 | 10.2098 | 5.916101 | 21.62196 | 18.68482 |
| P19137 | Lama1 | 0.173712 | 0.092522 | 0.276522 | 0.358174 |
| Q60675 | Lama2 | 2.291096 | 1.928191 | 2.082319 | 2.360602 |
| P97927 | Lama4 | 3.248685 | 1.496896 | 1.94127 | 2.605724 |
| Q61001 | Lama5 | 5.657156 | 3.221417 | 3.443519 | 4.489114 |
| P02469 | Lamb1 | 3.256893 | 2.31334 | 2.959571 | 3.440172 |
| Q61292 | Lamb2 | 7.054142 | 4.359017 | 3.978177 | 4.664288 |
| Q61087 | Lamb3 | 0.127379 | 0.128209 | 0.065401 | 0.10661 |
| F8VQJ3 | Lamc1 | 6.976295 | 4.334172 | 4.710444 | 7.252703 |

Table S5 Key Reagent Information

| Product Name | Manufacturer |
| --- | --- |
| Donkey Serum for Blocking | Wuhan Sanying, China |
| PV9001 Immunohistochemistry Kit | ZSGB-BIO, China |
| PV9003 Immunohistochemistry Kit | ZSGB-BIO, China |
| DAPI | Solarbio, China |
| Masson's Trichrome Staining Kit | Solarbio, China |
| HE Staining Kit | Solarbio, China |
| RNAeasy™ Animal RNA Isolation Kit (Spin Column) | Beyotime, China |
| Reverse Transcription Kit | Vazyme, China |
| ChamQ Universal SYBR qPCR Master Mix | Vazyme, China |
| RIPA Lysis Buffer | Solarbio, China |
| Fibronectin | Solarbio, China |
| Mouse Tail Collagen Type I | Solarbio, China |
| Fibroblast Medium | Vessal, China |
| Growth Differentiation Factor-15 (GDF-15) | ACROBiosystems, China |
| Sodium Deoxycholate | Solarbio, China |
| 0.5 M EDTA (pH 8.0) | Solarbio, China |
| 1 M Tris-HCl (pH 8.8) | Solarbio, China |
| 1 M Tris-HCl Buffer (pH 8.0) | Solarbio, China |
| Iodoacetic Acid | Sigma, USA |
| N-Ethylmaleimide (NEM) | Solarbio, China |
| 5X Protein Loading Buffer (with DTT) | Solarbio, China |
| Sodium Dodecyl Sulfate (SDS) | Solarbio, China |
| PG110 Fast PAGE Gel Preparation Kit (6%) | Yeasen Biotechnology, China |
| M5 SuperRange Prestained Protein Ladder (10-310 kDa) | Monad, China |
| BeyoECL Plus (Ultra-Sensitive ECL Kit) | Beyotime, China |
| Human Fibronectin ELISA Kit | NeoBioscience, China |

Table S6 TOP GO enrichment of in TNBS induced fibrotic intestine exposed to pUR4, compared to untreated TNBS induced fibrotic intestine

| id | Term | Category | ListHits | p-value | Enrichment_score |
| --- | --- | --- | --- | --- | --- |
| GO:0050853 | B cell receptor signaling pathway | biological_process | 10 | 1.61E-09 | 13.93333 |
| GO:0009617 | response to bacterium | biological_process | 17 | 1.87E-08 | 5.414095 |
| GO:0002376 | immune system process | biological_process | 30 | 4.47E-08 | 3.113594 |
| GO:0042102 | positive regulation of T cell proliferation | biological_process | 11 | 9.40E-08 | 8.174222 |
| GO:0006935 | chemotaxis | biological_process | 14 | 1.14E-07 | 5.911111 |
| GO:0030890 | positive regulation of B cell proliferation | biological_process | 9 | 1.31E-07 | 10.67234 |
| GO:0042113 | B cell activation | biological_process | 8 | 1.95E-07 | 12.38519 |
| GO:0042098 | T cell proliferation | biological_process | 10 | 3.54E-07 | 8.196078 |
| GO:0042832 | defense response to protozoan | biological_process | 8 | 6.91E-07 | 10.61587 |
| GO:0006954 | inflammatory response | biological_process | 22 | 1.26E-06 | 3.278431 |
| GO:0009897 | external side of plasma membrane | cellular_component | 41 | 6.23E-16 | 4.533862 |
| GO:0005615 | extracellular space | cellular_component | 79 | 9.00E-16 | 2.638067 |
| GO:0005576 | extracellular region | cellular_component | 79 | 9.43E-15 | 2.527516 |
| GO:0005886 | plasma membrane | cellular_component | 152 | 4.10E-13 | 1.69871 |
| GO:0009986 | cell surface | cellular_component | 44 | 1.03E-11 | 3.201393 |
| GO:0016020 | membrane | cellular_component | 190 | 2.33E-08 | 1.376489 |
| GO:0001772 | immunological synapse | cellular_component | 9 | 1.31E-07 | 10.67234 |
| GO:0031012 | extracellular matrix | cellular_component | 15 | 2.83E-05 | 3.512605 |
| GO:0042613 | MHC class II protein complex | cellular_component | 4 | 3.05E-05 | 20.26667 |
| GO:0098552 | side of membrane | cellular_component | 10 | 0.000223 | 3.980952 |
| GO:0016493 | C-C chemokine receptor activity | molecular_function | 6 | 3.28E-06 | 13.93333 |
| GO:0005125 | cytokine activity | molecular_function | 15 | 1.46E-05 | 3.715556 |
| GO:0008083 | growth factor activity | molecular_function | 12 | 1.53E-05 | 4.518919 |
| GO:0005178 | integrin binding | molecular_function | 12 | 1.87E-05 | 4.429139 |
| GO:0004950 | chemokine receptor activity | molecular_function | 5 | 2.91E-05 | 13.26984 |
| GO:0023026 | MHC class II protein complex binding | molecular_function | 4 | 4.51E-05 | 18.57778 |
| GO:0019957 | C-C chemokine binding | molecular_function | 5 | 4.67E-05 | 12.11594 |
| GO:0043395 | heparan sulfate proteoglycan binding | molecular_function | 5 | 5.81E-05 | 11.61111 |
| GO:0008467 | [heparan sulfate]-glucosamine 3-sulfotransferase 1 activity | molecular_function | 3 | 0.00019 | 23.88571 |
| GO:0004888 | transmembrane signaling receptor activity | molecular_function | 12 | 0.000319 | 3.294581 |

Table S7 TOP GO enrichment of in TNBS induced fibrotic intestine exposed to ATN161, compared to untreated TNBS induced fibrotic intestine

| id | Term | Category | ListHits | p-value | Enrichment_score |
| --- | --- | --- | --- | --- | --- |
| GO:0009617 | response to bacterium | biological_process | 17 | 9.11E-16 | 15.49836 |
| GO:0006629 | lipid metabolic process | biological_process | 18 | 6.76E-07 | 3.950145 |
| GO:0007586 | digestion | biological_process | 4 | 7.02E-07 | 53.18066 |
| GO:0055085 | transmembrane transport | biological_process | 14 | 7.51E-07 | 5.053366 |
| GO:0006953 | acute-phase response | biological_process | 4 | 4.07E-05 | 20.58606 |
| GO:0016042 | lipid catabolic process | biological_process | 6 | 7.89E-05 | 8.471256 |
| GO:0050873 | brown fat cell differentiation | biological_process | 4 | 0.000125 | 15.56507 |
| GO:0019433 | triglyceride catabolic process | biological_process | 3 | 0.000127 | 29.91412 |
| GO:0015813 | L-glutamate transmembrane transport | biological_process | 3 | 0.000183 | 26.59033 |
| GO:0006811 | monoatomic ion transport | biological_process | 12 | 0.000399 | 3.19084 |
| GO:0005576 | extracellular region | cellular_component | 39 | 9.05E-13 | 3.571835 |
| GO:0005615 | extracellular space | cellular_component | 33 | 2.00E-09 | 3.154515 |
| GO:0005811 | lipid droplet | cellular_component | 7 | 6.79E-06 | 9.971374 |
| GO:0034364 | high-density lipoprotein particle | cellular_component | 4 | 3.56E-05 | 21.27226 |
| GO:0098552 | side of membrane | cellular_component | 6 | 0.000255 | 6.837514 |
| GO:0009986 | cell surface | cellular_component | 14 | 0.000326 | 2.915911 |
| GO:0034361 | very-low-density lipoprotein particle | cellular_component | 3 | 0.000631 | 17.72689 |
| GO:0034366 | spherical high-density lipoprotein particle | cellular_component | 2 | 0.001364 | 35.45377 |
| GO:0031526 | brush border membrane | cellular_component | 4 | 0.001767 | 7.782536 |
| GO:0042589 | zymogen granule membrane | cellular_component | 2 | 0.004417 | 19.94275 |
| GO:0005179 | hormone activity | molecular_function | 8 | 1.38E-06 | 10.04989 |
| GO:0004806 | triglyceride lipase activity | molecular_function | 4 | 5.31E-06 | 33.58779 |
| GO:0016298 | lipase activity | molecular_function | 3 | 0.000127 | 29.91412 |
| GO:0005102 | signaling receptor binding | molecular_function | 9 | 0.00099 | 3.571835 |
| GO:0051428 | peptide hormone receptor binding | molecular_function | 2 | 0.001698 | 31.9084 |
| GO:0048019 | receptor antagonist activity | molecular_function | 2 | 0.002066 | 29.00763 |
| GO:0005509 | calcium ion binding | molecular_function | 11 | 0.002124 | 2.776838 |
| GO:0015250 | water channel activity | molecular_function | 2 | 0.00247 | 26.59033 |
| GO:0039706 | co-receptor binding | molecular_function | 2 | 0.00247 | 26.59033 |
| GO:0015386 | potassium:proton antiporter activity | molecular_function | 2 | 0.002907 | 24.54492 |

Table S8 TOP KEGG enrichment of in TNBS induced fibrotic intestine exposed to pUR4, compared to untreated TNBS induced fibrotic intestine

| id | Term | Classification_level1 | Classification_level2 | ListHits | p-value | Enrichment_score |
| --- | --- | --- | --- | --- | --- | --- |
| mmu04640 | Hematopoietic cell lineage | Organismal Systems | Immune system | 16 | 1.01E-10 | 7.995563 |
| mmu04060 | Cytokine-cytokine receptor interaction | Environmental Information Processing | Signaling molecules and interaction | 25 | 4.07E-09 | 3.922738 |
| mmu05321 | Inflammatory bowel disease | Human Diseases | Immune disease | 11 | 6.30E-08 | 8.290482 |
| mmu04672 | Intestinal immune network for IgA production | Organismal Systems | Immune system | 9 | 2.17E-07 | 9.851676 |
| mmu05310 | Asthma | Human Diseases | Immune disease | 7 | 5.23E-07 | 13.40923 |
| mmu05140 | Leishmaniasis | Human Diseases | Infectious disease: parasitic | 10 | 2.10E-06 | 6.66297 |
| mmu04658 | Th1 and Th2 cell differentiation | Organismal Systems | Immune system | 11 | 2.58E-06 | 5.812867 |
| mmu05330 | Allograft rejection | Human Diseases | Immune disease | 9 | 2.84E-06 | 7.388757 |
| mmu04610 | Complement and coagulation cascades | Organismal Systems | Immune system | 11 | 5.01E-06 | 5.437843 |
| mmu04662 | B cell receptor signaling pathway | Organismal Systems | Immune system | 10 | 7.37E-06 | 5.819556 |
| mmu04659 | Th17 cell differentiation | Organismal Systems | Immune system | 11 | 1.49E-05 | 4.862686 |
| mmu05145 | Toxoplasmosis | Human Diseases | Infectious disease: parasitic | 11 | 2.13E-05 | 4.682587 |
| mmu04514 | Cell adhesion molecules | Environmental Information Processing | Signaling molecules and interaction | 14 | 2.63E-05 | 3.677959 |
| mmu04940 | Type I diabetes mellitus | Human Diseases | Endocrine and metabolic disease | 8 | 6.04E-05 | 5.83803 |
| mmu05323 | Rheumatoid arthritis | Human Diseases | Immune disease | 8 | 0.00054 | 4.276697 |
| mmu04512 | ECM-receptor interaction | Environmental Information Processing | Signaling molecules and interaction | 8 | 0.00068 | 4.132538 |
| mmu05320 | Autoimmune thyroid disease | Human Diseases | Immune disease | 7 | 0.000923 | 4.469742 |
| mmu04062 | Chemokine signaling pathway | Organismal Systems | Immune system | 12 | 0.000924 | 2.88845 |
| mmu05340 | Primary immunodeficiency | Human Diseases | Immune disease | 5 | 0.001008 | 6.385346 |
| mmu05150 | Staphylococcus aureus infection | Human Diseases | Infectious disease: bacterial | 9 | 0.001117 | 3.477062 |

Table S9 TOP KEGG enrichment of in TNBS induced fibrotic intestine exposed to ATN161, compared to untreated TNBS induced fibrotic intestine

| id | Term | Classification_level1 | Classification_level2 | ListHits | p-value | Enrichment_score |
| --- | --- | --- | --- | --- | --- | --- |
| mmu03320 | PPAR signaling pathway | Organismal Systems | Endocrine system | 6 | 2.81E-05 | 9.95874 |
| mmu04972 | Pancreatic secretion | Organismal Systems | Digestive system | 6 | 0.000108 | 7.843609 |
| mmu04975 | Fat digestion and absorption | Organismal Systems | Digestive system | 4 | 0.000176 | 14.0687 |
| mmu04923 | Regulation of lipolysis in adipocytes | Organismal Systems | Endocrine system | 4 | 0.000574 | 10.36641 |
| mmu00561 | Glycerolipid metabolism | Metabolism | Lipid metabolism | 4 | 0.00084 | 9.379131 |
| mmu00910 | Nitrogen metabolism | Metabolism | Energy metabolism | 2 | 0.005743 | 17.37898 |
| mmu04974 | Protein digestion and absorption | Organismal Systems | Digestive system | 4 | 0.00603 | 5.47116 |
| mmu04977 | Vitamin digestion and absorption | Organismal Systems | Digestive system | 2 | 0.011306 | 12.31011 |
| mmu04924 | Renin secretion | Organismal Systems | Endocrine system | 3 | 0.014112 | 5.908852 |
| mmu04080 | Neuroactive ligand-receptor interaction | Environmental Information Processing | Signaling molecules and interaction | 7 | 0.015911 | 2.644627 |
| mmu00140 | Steroid hormone biosynthesis | Metabolism | Lipid metabolism | 3 | 0.024901 | 4.765204 |
| mmu04976 | Bile secretion | Organismal Systems | Digestive system | 3 | 0.030019 | 4.431639 |
| mmu04514 | Cell adhesion molecules | Environmental Information Processing | Signaling molecules and interaction | 4 | 0.030438 | 3.376487 |
| mmu04930 | Type II diabetes mellitus | Human Diseases | Endocrine and metabolic disease | 2 | 0.041669 | 6.155055 |
| mmu04724 | Glutamatergic synapse | Organismal Systems | Nervous system | 3 | 0.041793 | 3.887403 |
| mmu04979 | Cholesterol metabolism | Organismal Systems | Digestive system | 2 | 0.044867 | 5.908852 |
| mmu02010 | ABC transporters | Environmental Information Processing | Membrane transport | 2 | 0.048153 | 5.681589 |
| mmu04152 | AMPK signaling pathway | Environmental Information Processing | Signal transduction | 3 | 0.054513 | 3.48948 |
| mmu05330 | Allograft rejection | Human Diseases | Immune disease | 2 | 0.054978 | 5.275761 |
| mmu05332 | Graft-versus-host disease | Human Diseases | Immune disease | 2 | 0.054978 | 5.275761 |
